# Identification and Analysis of lncRNA and circRNA Related to Wheat Grain Development

**DOI:** 10.3390/ijms25105484

**Published:** 2024-05-17

**Authors:** Meng Wang, Lu Wang, Shuanghong Wang, Junli Zhang, Zhe Fu, Panpan Wu, Anqi Yang, Dexiang Wu, Genlou Sun, Chengyu Wang

**Affiliations:** 1 College of Agronomy, Anhui Agricultural University, Hefei 230036, China15955162171@163.com (A.Y.); wangcy523@163.com (C.W.); 2 Biology Department, Saint Mary’s University, Halifax, NS B3H 3C3, Canada

**Keywords:** lncRNA, circRNA, wheat grain, miRNA, regulatory gene

## Abstract

The role of lncRNA and circRNA in wheat grain development is still unclear. The objectives of this study were to characterize the lncRNA and circRNA in the wheat grain development and to construct the interaction network among lncRNA, circRNA, and their target miRNA to propose a lncRNA–circRNA–miRNA module related to wheat grain development. Full transcriptome sequencing on two wheat varieties (Annong 0942 and Anke 2005) with significant differences in 1000-grain weight at 10 d (days after pollination), 20 d, and 30 d of grain development were conducted. We detected 650, 736, and 609 differentially expressed lncRNA genes, and 769, 1054, and 1062 differentially expressed circRNA genes in the grains of 10 days, 20 days and 30 days after pollination between Annong 0942 and Anke 2005, respectively. An analysis of the lncRNA–miRNA and circRNA–miRNA targeting networks reveals that circRNAs exhibit a more complex and extensive interaction network in the development of cereal grains and the formation of grain shape. Central to these interactions are tae-miR1177, tae-miR1128, and tae-miR1130b-3p. In contrast, lncRNA genes only form a singular network centered around tae-miR1133 and tae-miR5175-5p when comparing between varieties. Further analysis is conducted on the underlying genes of all target miRNAs, we identified TaNF-YB1 targeted by tae-miR1122a and TaTGW-7B targeted by miR1130a as two pivotal regulatory genes in the development of wheat grains. The quantitative real-time PCR (qRT-PCR) and dual-luciferase reporter assays confirmed the target regulatory relationships between miR1130a-TaTGW-7B and miR1122a-TaNF-YB1. We propose a network of circRNA and miRNA-mediated gene regulation in the development of wheat grains.

## 1. Introduction

Wheat (*Triticum aestivum* L.) is the most widely planted crop globally [1]. Wheat provides about 35% of the world’s population with essential carbohydrates every day. With the continuous increase in the world population, the demand for wheat around the world is also constantly increasing. However, adverse factors such as precipitation, heat waves, and other extreme factors, as well as changes in pests, diseases, and pathogens, have limited wheat production. It is estimated that current grain crop yields will need to double in thirty years to meet the population growth’s demand for food. The yield of wheat is mainly determined by the number of ears per unit area, the number of grains per ear, and the weight of 1000 grains. Studying the internal mechanisms of wheat grain development is of great significance for improving wheat yield. At present, molecular cloning research on wheat grain weight related genes is limited, but map-based cloning provides a large number of quantitative trait loci (QTLs), including TaGW2 [2], TaTGW-7A [3], TaGW8 [4], TaGW7 [5], TaGS5-3A [6], etc. Research on grain weight QTLs shows that, although many QTLs have been identified, the impact of a single QTL on grain weight is usually small, with an average increase in grain weight not exceeding 10%. The smaller effect of these QTLs is partly due to the polyploid nature of wheat, which has high gene redundancy. The variation of a single gene is often masked by the function of other homoeologous genes. Although the mechanism of regulating grain size is conserved in cereal crops, the mechanism of increased grain filling is still elusive [7]. The development process of wheat grains determines the weight and size of wheat grains, and directly affects the yield and quality of wheat [8,9,10]. The research on the regulation mechanism of wheat grain development has a great effect on increasing the wheat yield and improving the wheat quality. There are many studies on the regulatory mechanisms of wheat grain development. Wang et al. [11] found that TaGS2 may have a negative regulatory effect on the 1000-grain weight of wheat. On the contrary, it was found that the overexpression of TaKNOX1s leads to increased grain size, increased seed size, but decreased tillering. The reduced expression of TaKNOX1s leads to a decrease in grain weight, indicating that TaKNOX1s has a positive regulatory effect on the 1000-grain weight of wheat. TaWG2 in wheat was reported to be related to the 1000-grain weight, and two single nucleotide polymorphisms (SNPs) were detected in the promoter region of TaWG2, both of which affect the grain weight and width of wheat grains [2]. Cristina et al. [12] found 10 genes associated with the 1000-grain weight of wheat, namely TaSST-D1, TaFlo2-A1, Ppd-D1, 6-SFT-A2, TaTEF-7A, Tackx4, TaSnRK2.3-1B, 1-FEH w3, TaGS-D1, and TaPPH-7A genes. The overexpression of TaDA1 in wheat also reduces the size and weight of wheat grains, while downregulation leads to the opposite result. Liu et al. [13] found that there is an interaction between TaGW2 and TaDA1. The additive effect of TaDA1 and TaGW2 on the 1000-grain weight of wheat can be achieved by simultaneously downregulating TaDA1 and TaGW2 [2,13]. TaCwi cell wall invertase (CWI) is a key enzyme in tissue development and carbon allocation, and has a high correlation with grain weight [14]. In addition, studies have shown that chromosome 7P can stably and significantly increase the grain weight by about 2 g at different grain weight levels in different wheat backgrounds [15]. TaMADS29 gene has a positive regulatory effect on wheat grains [16]. Auxin signaling is crucial for the development of grain size and weight. The TAIAA21 mutation significantly increased the grain length, width, and 1000-grain weight, and can act as a negative regulator of grain size and weight development through the ARF25-ERFs module, thereby increasing wheat yield [17].

Many studies have shown that microRNAs play a crucial regulatory role in wheat grain development. Meng et al. [18] identified 104 miRNAs related to grain filling, of which 86 may be related to the control of grain filling, and 18 may be related to the maturation process. They found that seven miRNAs were significantly upregulated at 15 and 25 days after pollination and reached their highest levels at 30 days, while the expression of miR1852 was inhibited. miR156, miR396, miR160, miR172, and miR164 may play important regulatory roles in wheat grains [19]. miR396 not only participates in the regulation of various abiotic stresses, but also regulates the development of wheat grains [19,20,21,22,23]. Han et al. [24] found that four known miRNA families (miR169, miR166, miR164, and miR160) and 22 new miRNAs are involved in the regulation of wheat seed development and metabolism. In developing seeds, miR164 and miR160 increase, while miR169 decreases from 5 DAP to 20 DAP.

LncRNA is a long-chain noncoding RNA that does not encode proteins and primarily exerts its biological functions through mechanisms such as cell cycle regulation, mRNA degradation, and increased mRNA stability [25]. LncRNA can affect the growth and development of plants. It was found that the natural antisense transcriptional lncRNA produced by MADS and MAS affects the cold-induced FLOWERING4 (MAF4) site and is induced by cold. lncRNA MAS fine-tune the flowering time by regulating the expression of MAF4, a member of the FLC family. lncRNA is associated with pollen development in Chinese cabbage and programmed cell death in rice anthers [26,27,28], and can affect the ripening of wild tomatoes, whilst silencing lncRNA1459 and lncRNA1840 can lead to the delayed ripening of wild tomatoes, and CRISPR/cas9-mediated lncRNA1459 mutations significantly inhibit tomato fruit ripening [29,30]. These results indicated that although lncRNA does not encode proteins, it plays an important regulatory role in plant growth and development.

CircRNA is an important supplement to noncoding RNA, are mainly derived through the fusion of downstream 3′ splicing donors and upstream 5′ splicing receptors through a non-standard alternative splicing mechanism of reverse splicing [31]. At present, some functions of circRNA have been extensively studied in animals, but are still limited in plants. According to current predictive tools, few identified circRNAs have miRNA binding sites [32,33,34]. Due to the presence of binding proteins such as AGO, RNA polymerase II, MBL, and QKI, CircRNA also has the ability to act as a protein sponge [35,36,37,38,39]. It was predicted that a large number of animal circRNAs have the ability to encode proteins, indicating that they can be translated into proteins [40,41,42,43]. Xu et al. (2019) found that the expression levels of some circRNAs showed significant differences in wheat varieties with different root phenotypes and successfully identified 10 differentially expressed genes involved in wheat root length regulation, indicating that circRNAs may also play a potential role in regulating wheat root length. Wu et al. [44] found that circRNA in wheat is more abundant than that in other plants, and constructed a circRNA mediated network related to tillering and that 7 circRNAs are associated with known tillering/branching genes in rice or Arabidopsis. Chen et al. [45] revealed a possible connection between circRNA regulation and the expression of functional genes related to rice grain development mediated by light signals.

LncRNA and circRNA are regulated by miRNA, and can act as sponges for miRNA, thereby regulating the transcription and translation of downstream target genes. At present, there have been some studies on the interaction mechanism between lncRNA and miRNA, and circRNA and miRNA in plants. Li et al. [46] identified lncRNA genes related to drought stress and explored the response of lncRNA–miRNA modules in wheat drought stress, revealing the regulatory role of lncRNA–miRNA in drought tolerance regulatory networks. Li et al. [47] found that wheat circRNA has significant variety specificity and identified 239 wheat circRNAs responsive to drought stress and 5 potential circRNA–miRNA-mRNA regulatory modules. LncRNA indirectly regulates the expression of CSD1 by competitively binding to miR398, thereby affecting Dn398’s resistance to cold. Previous research has demonstrated the important role of the interaction mechanism of lncRNA circRNA miRNA in crop growth, development, and stress resistance activities [48].

In recent years, with the development of technologies, various technical methods, including transcriptome analysis, have been used to study the molecular mechanisms of wheat grain development. However, the role of lncRNA and circRNA in wheat grain development is still unclear. The objectives of this study were to characterize the lncRNA and circRNA in the wheat grain development in two wheat varieties (Annong 0942 and Anke 2005) with a significant difference in the 1000-grain weight through full transcriptome sequencing; and to construct the interaction network among lncRNA, circRNA, and their target miRNA to propose an lncRNA- circRNA- miRNA module related to wheat grain development. Our results will generate novel knowledge on understanding wheat grain development.

## 2. Results

### 2.1. Statistical Analysis of Grain-Related Traits

To ensure the accuracy of the analysis of lncRNA and circRNA in the development of wheat grains, we selected and evaluated two Chinese wheat varieties that exhibit significant differences in the trait of 1000-grain weight (TGW). The varieties in question are the large-grain wheat cultivar AnNong 0942 and the small-grain wheat cultivar AnKe 2005, both of which are locally bred in the Huang-Huai region of China. The 1000-grain weight of wheat variety Annong0942 at 10 d, 20 d, and 30 d after flowering was 52 g, 102 g, and 91 g, respectively. The 1000-grain weight of wheat at maturity was 59 g. The 1000-grain weight of the wheat variety Anke 2005 at 10 d, 20 d, and 30 d after flowering was 23 g, 59 g, and 46 g, respectively. The 1000-grain weight of the mature Anke 2005 wheat was 39 g. Statistical analysis showed a highly significant difference in the 1000-grain weight between the two wheat varieties at 10 d, 20 d, and 30 d after flowering, and maturity (Figure S1c). The 1000-grain weight at 20 d after flowering increased significantly compared to that at 10 d after flowering, and also significantly decreased at 30 days after flowering compared to the 1000-grain weight at 20 days after flowering in each variety (Figure S1e). The grain length and width of Annong 0942 and Anke 2005 showed significant differences (Figure S1d).

### 2.2. Identification and Prediction of lncRNA Genes and circRNAs

Considering the pivotal role of lncRNA genes and circRNAs in plant growth and development, we embarked on Illumina sequencing to identify key candidate non-coding RNAs during the development of wheat grains. Following the exclusion of redundant and low-quality reads, we identified a total of 3620 (Figure S2) long non-coding RNAs (lncRNA genes) through whole transcriptome sequencing, which included antisense/exonic, intronic, intergenic downstream, and intergenic upstream lncRNA genes (Figure S3). Additionally, we identified a total of 2320 circular RNAs (circRNAs), comprising intergenic, intronic, antisense, exonic, and sense-overlapping types (Figure S4). A minority of lncRNA genes (4.78%) were classified as intronic, while the rest ranged from 5.33% to 19.31% across other types. The majority of circRNAs were categorized as intergenic (36%), followed by antisense circRNAs (25%), with a minority (4%) being intronic (Figure S5). Furthermore, the majority of lncRNA genes (33.89%) were between 200 and 400 bp in length (Figure S6), and approximately 8.62% of circRNAs exceeded 2000 bp (Figure S7), aligning with the characteristics of lncRNA genes and circRNAs observed in other species.

### 2.3. Identification of Differentially Encoded Genes and Statistical Identification of Differentially Non-Coding Genes

**Figure 1:**
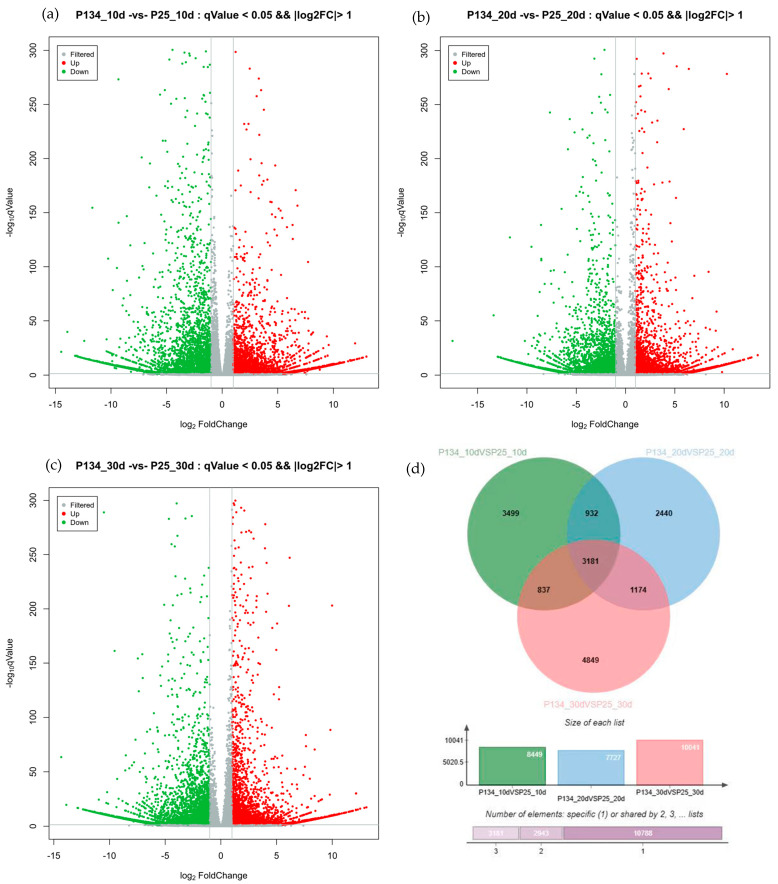
Analysis of significant mRNA differentially expressed genes during wheat grain development. (**a**) P134_10 vs. P25_ Volcano map of significantly differentially expressed genes; (**b**) P134_20 vs. P25_20 significantly differentially expressed gene volcanic maps; (**c**) P134_30 vs. P25_ 30 significantly differentially expressed gene volcanograms; (**d**) Wayne map of differentially expressed genes at different stages.

In order to understand the impact of gene differential expression on wheat development, we compared and analyzed the differentially expressed genes in two different wheat varieties, Annong 0942 and Anke 2005. By calculating and screening the significantly differentially expressed genes at different stages of wheat grain development (Figure 1), we finally found 8449 differentially expressed genes in the mRNA of wheat varieties Annong 0942 and Anke 2005 10 days after pollination, among which 4121 were upregulated and 4328 were downregulated. There were 7727 mRNA differentially expressed genes in the wheat grains of 20 days after pollination between Annong 0942 and Anke 2005, of which 3469 mRNA differentially expressed genes were upregulated and 4258 were downregulated. There were total of 10,041 differentially expressed mRNA genes in the wheat grains of 30 days after pollination between Annong 0942 and Anke 2005, among which 4497 were upregulated and 5544 were downregulated. A total of 3181 common significantly differentially expressed genes were found in different periods.

**Figure 2:**
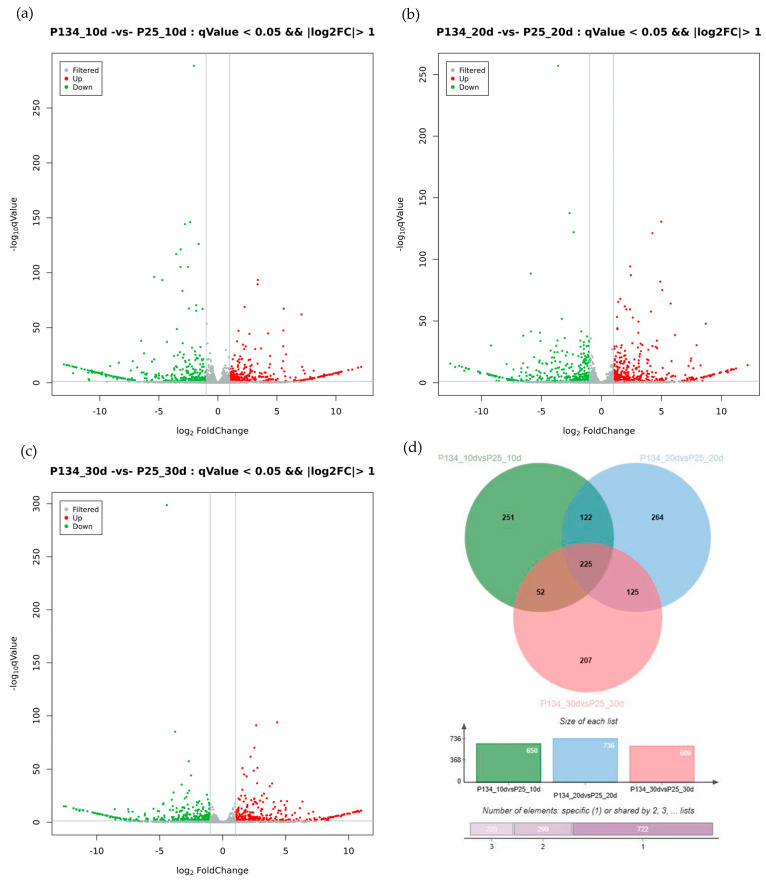
Analysis of significant lncRNA differentially expressed genes during wheat grain development. (**a**) lncRNA volcano plot of P 134_10 vs. P 25_10; (**b**) lncRNA volcano plot of P 134 _ 20 vs. P 25_20; (**c**) lncRNA volcano plot of P 134_30 vs. P 25_30; (**d**) Wayne diagram of differentially expressed lncRNA genes in different periods.

Analysis of the differential expression of lncRNA genes in different developmental stages of wheat revealed 650 differentially expressed lncRNA genes in the grains of 10 days after pollination between Annong 0942 and Anke 2005, among which 338 were upregulated and 318 were downregulated (Figure 2). In the grain of 20 days after pollination, a total of 736 differentially expressed genes were detected between Annong 0942 and Anke 2005, among which 392 were upregulated and 344 were downregulated. In the grain of 30 days after pollination, there were 609 differentially expressed lncRNA genes between Annong 0942 and Anke 2005, with 298 genes upregulated and 311 genes downregulated. Venn plot analysis revealed a total of 225 common and significantly differentially expressed lncRNA genes in the wheat grains of different grain types at different stages.

**Figure 3:**
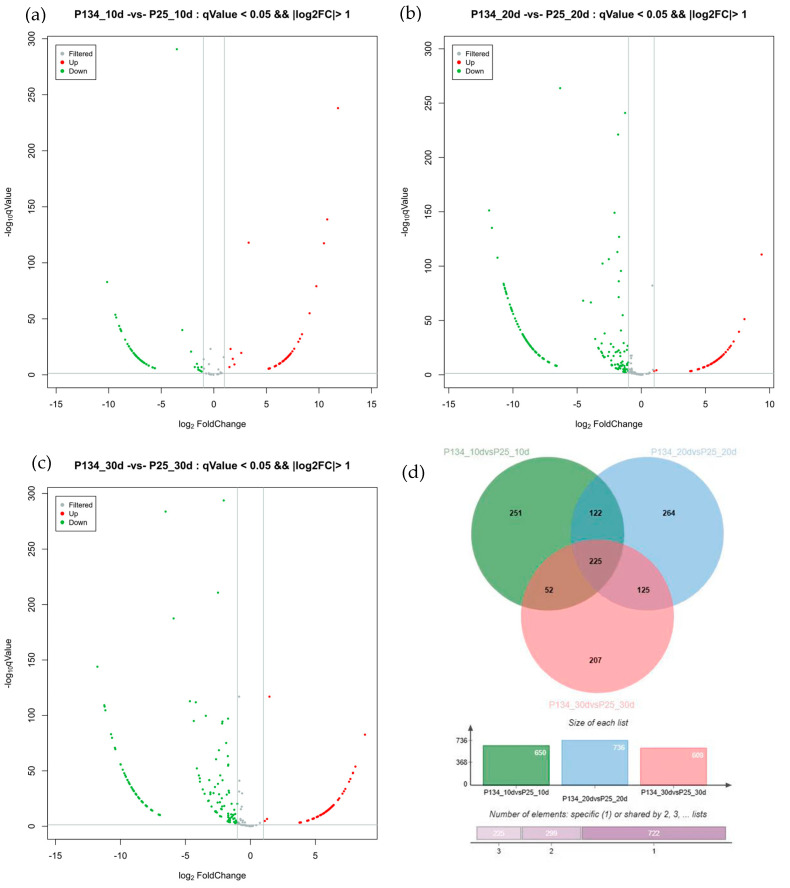
Analysis of a significant circRNA differentially expressed genes during wheat grain development: (**a**) Volcano plot of P134_10 vs. P25_10; (**b**) P134_20 vs. P25_20; (**c**) P134_30 vs. P25_30; and (**d**) Wayne diagram of differentially expressed genes in different periods.

There were 769 differentially expressed circRNA genes detected in the grains of 10 days after pollination between Annong 0942 and Anke 2005 (Figure 3), among which 405 genes were upregulated and 364 genes were downregulated. In the grain of 20 days after pollination, a total of 1054 differentially expressed circRNA genes were found between Annong 42 and Anke 2005, among which 454 were upregulated and 600 were downregulated. In the grain of 30 days after pollination, there were a total of 1062 differentially expressed circRNA genes between Annong 0942 and Anke 2005, with 516 genes upregulated and 546 genes downregulated. The Venn plot analysis of significantly differentially expressed circRNA genes in the wheat grains of different grain types at different stages revealed a total of 78 common significantly differentially expressed circRNA genes.

### 2.4. GO Enrichment and KEGG Enrichment Analysis of Differentially Expressed mRNA Genes

We described the function and metabolic pathways of mRNA differentially expressed genes by conducting GO enrichment and KEGG enrichment analysis on significantly differentially expressed genes in wheat development grains. The differentially expressed genes in the 10 days after the pollination grains of Annong 0942 and Anke 2005 were mainly enriched in protein complex polymers (GO: 0051259), reactive oxygen species (GO: 000302), and hydrogen peroxide (GO: 0042542) in biological processes. In cell composition, they were mainly enriched in components such as nucleosomes (GO: 000786), lipid droplets (GO: 0005811), and extracellular domains (GO: 0005576). In terms of molecular function, they were mainly enriched in functions such as ADP binding (GO: 0043531), phenylalanine ammonia lyase activity (GO: 0045548), and protein self-association (GO: 0043621). KEGG enrichment analysis found that the differentially expressed mRNAs of Annong 0942 and Anke 2005 were mainly enriched in the interaction between plants and pathogens (taes04626), peroxisome (taes04146), and protein processing (taes04141) pathways in the endoplasmic reticulum (Figure S8).

The differentially expressed genes in the 20 days after pollination grains of Annong 0942 and Anke 2005 were mainly enriched in biological processes such as protein complex oligomerization (GO: 0051259), reactive oxygen species response (GO: 000302), and water response (GO: 0009415). In cellular composition, they were mainly enriched in the MCM complex (GO: 0042555), small subunit processing body (GO: 0032040), and nucleosome (GO: 000786), and bound to snoRNA (GO: 0030515) in pathways such as unfolded protein binding (GO: 0051082) and DNA helicase activity (GO: 0003678). The KEGG enrichment analysis found that differentially expressed genes were mainly enriched in pathways such as DNA replication (taes03030), eukaryotic ribosome biogenesis (taes03008), and protein processing in the endoplasmic reticulum (taes04141) (Figure S9).

The differentially expressed genes between Annong 0942_30 and Anke 2005_30 were mainly enriched in biological processes such as photosynthesis, light collection in Photosystem I (GO: 0009768), protein chromophore junction (GO: 0018298), and cinnamic acid biosynthesis (GO: 0009800). In cell components, they were mainly enriched in nucleosomes (GO: 0000786), Photosystem II (GO: 0009523), and plastid leaflets (GO: 001287). In terms of molecular function, they were mainly enriched in protein heterodimerization activity (GO: 0046982), chlorophyll binding (GO: 0016168), and phenylalanine ammonia lyase activity (GO: 0045548).

The KEGG enrichment of differentially expressed genes found that upregulated differentially expressed genes were significantly enriched in pathways such as protein processing (taes04141), phenylpropanoid biosynthesis (taes0940), and phenylalanine metabolism (taes0360) in the endoplasmic reticulum. The downregulated differentially expressed genes were significantly enriched in pathways such as carbon sequestration (taes00710), ethoxylate and dicarboxylic acid metabolism (taes00630), and photosynthesis (taes0195) in photosynthetic organisms (Figure S10). By comparing and analyzing the differentially expressed mRNA genes of Annong 0942 and Anke 2005 at 10, 20, and 30 days after pollination, we found that a large number of differentially expressed mRNA genes were enriched in pathways closely related to wheat growth and development, such as photosynthesis, protein processing, starch synthesis, and ADP binding. We speculated that these differentially expressed genes play an important role in the grain development of Annong 0942 and Anke 2005, leading to significant differences in grain length, width, and 1000-grain weight between the two wheat varieties.

### 2.5. GO Enrichment and KEGG Enrichment Analysis of Differentially Expressed lncRNA Genes

We analyzed differentially expressed lncRNA genes during wheat grain development through GO enrichment and KEGG enrichment, and described the functions of differentially expressed lncRNA genes. The GO enrichment results showed that 10 days after pollination, Annong 0942 and Anke 2005 were mainly enriched in defense reactions (GO: 0006952), reactions to cadmium ions (GO: 0046686), and cell wall tissues (GO: 0071555) in biological processes. In cellular composition, they were mainly enriched in splice complex (GO: 000243), U2 type fold precursor (GO: 0071004), and centrosome (GO: 005813). In molecular function, they were bound to ADP (GO: 0043531), the nutrient pool activity (GO: 0045735), and metal ion binding (GO: 0046872) are mainly enriched. KEGG enrichment analysis found that differentially expressed lncRNA genes were mainly enriched in methionine metabolism (ko03010), carotenoid biosynthesis (ko00906), and protein processing in the endoplasmic reticulum (ko04141) (Figure S11).

The GO enrichment results of differentially expressed lncRNA genes in large grain wheat Annong 0942 and small grain wheat Anke 2005 at 20 days after pollination showed that differentially expressed lncRNA genes were mainly enriched in biological processes such as carbohydrate metabolism (GO: 0005975), potassium ion transport (GO: 0006813), and response to water deficiency (GO: 0009414). They were mainly enriched in membrane forming bodies (GO: 0009524), microtubules (GO: 0005874), and centrosomes (GO: 0005813) in cell components, and mainly enriched in functions such as polygalacturonase activity (GO: 0004650), nutrient pool activity (GO: 0045735), and N, N-dimethylaniline monooxygenase activity (GO: 0004499) in terms of molecular function. The results of KEGG enrichment showed that the differentially expressed lncRNA genes of Annong 0942 and Anke 2005 at 20 days after pollination were mainly enriched in metabolic pathways such as lysine degradation (ko00310), cysteine and methionine metabolism (ko00270), and carotenoid biosynthesis (ko00906) (Figure S12).

The GO enrichment results of differentially expressed lncRNA between Annong 0942 and Anke 2005 at 30 days after pollination showed that differentially expressed lncRNA genes were mainly enriched in microtubule cytoskeleton tissue (GO: 000226), multicellular biological development (GO: 007275), and defense response signaling pathways (GO: 0010204) in biological processes, and mainly enriched in membrane forming bodies (GO: 0009524), the microtubule (GO: 0005874), and cytoplasmic large ribosome subunit (GO: 0022625) in cell components, and mainly enriched in molecular functions such as serine type endopeptidase inhibitor activity (GO: 0004867), protein serine/threonine kinase activity (GO: 0004674), and N, N-dimethylaniline monooxygenase activity (GO: 0004499). According to KEGG enrichment analysis, differentially expressed genes were mainly enriched in pathways such as cysteine and methionine metabolism (ko00270), the biosynthesis of keratin, cork, and wax (ko00073), and the interaction between plants and pathogens (ko04626) (Figure S13).

Through the analysis of differentially expressed lncRNA genes in Annong 0942 and Anke 2005, we found that although the number of differentially expressed lncRNA genes were significantly reduced compared to mRNA genes, they were still mainly enriched in functions related to energy synthesis and transportation, such as carbohydrate metabolism, transferase activity, ADP synthesis, and photosynthesis, In addition, we found that the differentially expressed lncRNA genes at 20 and 30 days after pollination in Annong 0942 and Anke 2005 were also enriched in functions related to cell structure, such as cytoskeleton, microfilaments, microtubules, and cell walls. These results indicated that lncRNA not only plays a key role in wheat energy metabolism, but also may play an important role in improving cell structure.

### 2.6. GO Enrichment and KEGG Enrichment Analysis of Differentially Expressed circRNA Genes

We described the function and metabolic pathways of circRNA differentially expressed genes by conducting GO enrichment and KEGG enrichment analysis on significantly differentially expressed genes in wheat grains during development. The differentially expressed genes between Annong 0942 and Anke 2005 at 10 days after pollination were mainly enriched in the resistance gene-dependent defense response signaling pathway (GO: 0009870), response to water deficiency (GO: 0009414), and actin filament tissue (GO: 0007015) in biological processes. They were mainly enriched in mitochondria (GO: 0005739), nucleosomes (GO: 000786), and plastids (GO: 0009536) in cellular components. The KEGG enrichment results showed that differentially expressed circRNAs were mainly enriched in metabolic pathways such as glyoxylate and dicarboxylic acid metabolism (ko00630), protein processing in the endoplasmic reticulum (ko04141), and ribosome (ko03010) (Figure S14).

The differentially expressed circRNA genes of Annong 0942 and Anke 2005 at 20 days after pollination were mainly enriched in biological processes such as carbohydrate storage (GO: 0052576), positive regulation of transcription, DNA template (GO: 0045893), and starch biosynthesis process (GO: 0019252), mainly enriched in cellular components such as starch granules (GO: 0043036), amyloplasts (GO: 0009501), and cytoskeleton (GO: 005856), and mainly enriched in nutrient pool activity (GO: 0045735), starch binding (GO: 2001070), and monosaccharide binding (GO: 0048029) in terms of molecular function. KEGG enrichment analysis revealed that their differentially expressed genes were mainly enriched in metabolic pathways such as starch and sucrose metabolism (ko00500), amino and nucleotide sugar metabolism (ko00520), and phosphatidylinositol signaling system (ko04070) (Figure S15).

The differentially expressed genes between Annong 0942 and Anke 2005 at 30 days after pollination were mainly enriched in biological processes such as carbohydrate storage (GO: 0052576), pollen development (GO: 0009555), and gene expression regulation (GO: 0010468), and mainly enriched in cellular composition of, e.g., starch granules (GO: 0043036), membranes (GO: 0016020), and extracellular space (GO: 0005615), and mainly enriched in terms of molecular function such as nutrient pool activity (GO: 0045735), monosaccharide binding (GO: 0048029), and IgE binding (GO: 0019863). The KEGG enrichment analysis found that differentially expressed circRNAs were mainly enriched in metabolic pathways such as the messenger RNA monitoring pathway (ko03015), amino and nucleotide sugar metabolism (ko00520), and phenylpropanoid biosynthesis (ko00940) (Figure S16). After analyzing the differentially expressed circRNA genes between Annong 0942 and Anke 2005, we found that although the number of differentially expressed circRNA genes were small, they have an important impact on plant growth and development. Although the functions enriched by differentially expressed circRNA genes were few, they were all related to growth and development, including monosaccharide binding, nutrient storage activity, starch binding, and play an important role in pollen development.

### 2.7. Construction of lncRNA circRNA miRNA Interaction Network

We commenced by employing TargetFinder to predict the target miRNAs of lncRNA genes and circRNAs, aiming to elucidate the miRNAs that interact with lncRNA genes and circRNAs (Table S1). Subsequently, we enriched and analyzed the total differential lncRNA genes and differential circRNAs, and constructed the interaction networks of lncRNA–miRNA and circRNA–miRNA targets using the R ‘network’ package (Figures S17–S25). The results indicated that the identified lncRNA genes, circRNAs, and miRNAs in both varieties could form extensive interaction networks. Within the lncRNA–miRNA interaction network, we observed that at different developmental stages (10 d, 20 d, 30 d) in both varieties, there were predominantly singular lncRNA–miRNA interaction networks centered around tae-miR1133 and tae-miR5175-5p. Interestingly, in the circRNA–miRNA interaction networks, circRNAs and miRNAs formed more complex networks, mainly connected through core miRNAs such as tae-miR1177, tae-miR1128, and tae-miR1130b-3p. In contrast, the lncRNA networks were more dispersed when comparing large and small grain types at different developmental stages of seed development, with many isolated interaction networks present and a similar core composition. Surprisingly, similarly to the variety comparisons, the circRNA networks formed during the different developmental stages of seed development in both large and small grain types were vast, suggesting that circRNAs play a significant role in seed development and grain size formation.

### 2.8. Prediction and qRT PCR Validation of Downstream Target Genes in Interaction Modules

**Figure 4:**
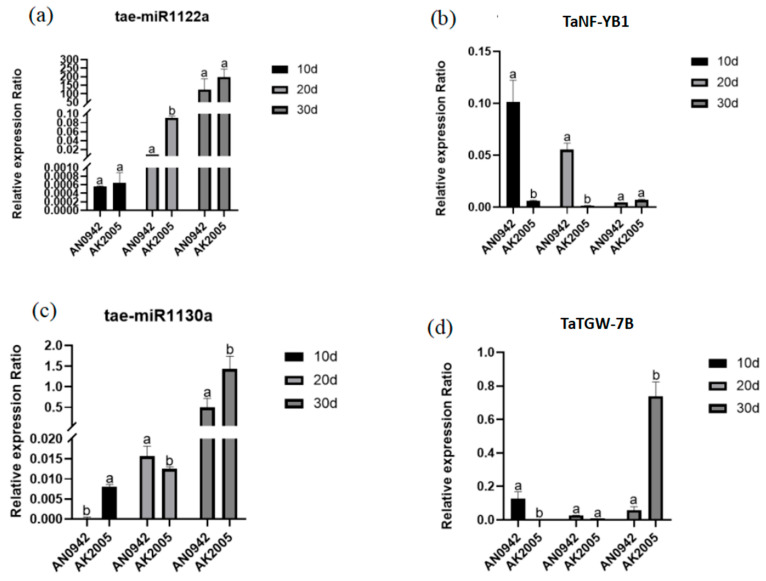
qRT PCR validation of miRNA and mRNA. (**a**,**b**) qRT PCR validation results of tae-miR1122a and its downstream gene TaNF-YB1; (**c**,**d**) qRT-PCR validation results of tae-miR1120b-3p and its downstream gene TaTGW-7B.one-way significance analysis was used, a and b indicate significant differences at the level of 0.05.

We further predicted and analyzed the predicted miRNA-related target genes using PSRobot. A total of 12,829 target genes for 84 miRNAs and further annotated their gene functions were predicted. We found that an important regulatory gene TaNF-YB1-D (TraesCS6D02G268200) and another gene TaTGW-7B (TraesCS7B03G0358400) regulate wheat grain development in the predicted downstream target genes. Previous research results have shown that knocking out TaNF-YB1-D in wheat led to abnormal early grain development, and TaTGW-7B also has a regulatory effect on wheat grain development, we selected TaNF-YB1-D and its upstream regulatory miRNA (tae-miR1122a), as well as TaTGW-7B and its upstream miRNA (tae-miR1130a) as our key and important regulatory modules for validation. The quantitative results showed that the expression of the target gene TraesCS6D02G268200 was significantly higher in the large grain wheat variety Annong 0942 at 10 and 20 days after pollination than in the small grain wheat variety Anke 2005, while the expression of miR1122a was lower in the large grain wheat variety at 10 and 20 days after pollination than in the small grain wheat variety; however, the expression was significantly lower in Annong 0942 at 20 days after pollination than in Anke 2005, which is opposite to the expression of the target gene. The expression level of TraesCS7B03G0358400 in Annong 0942 at 10 days after pollination was significantly higher than that in Anke 2005, while its upstream miRNA (tae-miR1130a) was significantly lower in Annong 0942 at 10 days after pollination than in Anke 2005 (Figure 4). Further analysis was conducted on one lncRNA and nine circRNAs that exhibit targeting relationships upstream of tae-miR1122a and tae-miR1130a. The lncRNA did not show a differential expression during seed development or between the varieties under comparison. However, circRNA_0957 and circRNA_1041, presumed to be upstream regulatory RNAs of tae-miR1122a, were expressed at higher levels during the early development of the large grain phenotype. Interestingly, circRNA_0587 and circRNA_2127, as putative upstream regulators of tae-miR1130a, also exhibited higher expression in the large grain phenotype in the intervariety comparisons, aligning with our expectations.

### 2.9. Analysis of the Experimental Results of Dual Luciferase

**Figure 5:**
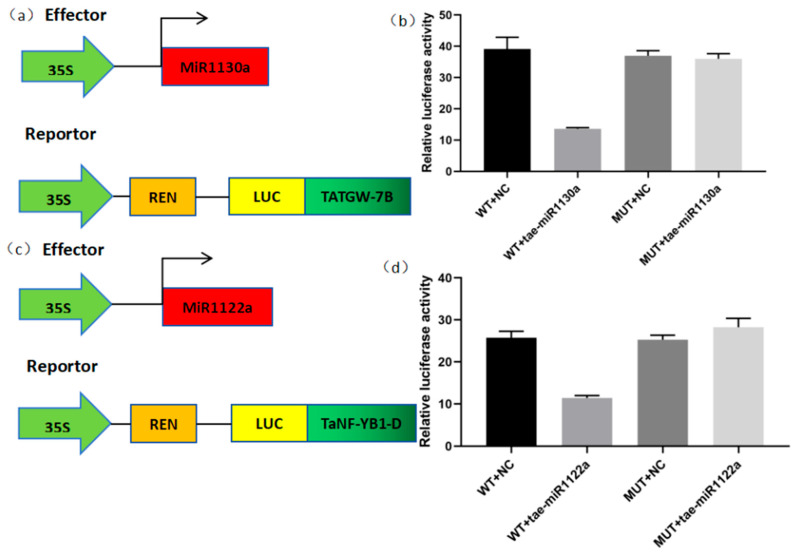
Analysis of dual luciferase activity. (**a**,**c**) Schematic diagram of the dual luciferase activity system carrier. (**b**,**d**) are the schematic diagram of the dual luciferase experiment results for the miR1130a targeting gene TATGW-7B and miR1122a targeting gene TaNF-YB1-D, respectively.

The pCAMBIA1300-miR1130a overexpression vector, WT-TaTGW-7B-pGreenII 0800-LUC, and MUT-TaTGW-7B-pGreenII 0800-LUC fusion vectors were, respectively, introduced into *Agrobacterium tumefaciens* and co-injected into tobacco leaves for co expression. The LUC enzyme activity at 48 h after tobacco injection was measured. The results showed that the LUC activity in the co-expression of miR1130a and TaTGW-7B was significantly weaker than that in miR1130a and LUC, and the LUC activity in the co-expression of miR1130a and TaTGW-7B-MUT showed that miR1130a could target and cleave TaTGW-7B. Furthermore, the targeting relationship between miR1122a and TaNF-YB1-D was verified through a dual luciferase system, and the LUC activity in the co-expression of miR1122a and TaNF-YB1-D was significantly weaker than that of miR1122a and LUC (Figure 5). The LUC activity in the co-expression of miR1122a and TaNF-YB1-D-MUT also indicated that miR1130a can target and cleave TaNF-YB1-D.

## 3. Materials and Methods

### 3.1. Plant Materials

Two wheat varieties (Annong 0942 and Anke 2005) were planted in the National High tech Agriculture Park of Anhui Agricultural University under natural environmental conditions according to normal field management. The flowering time for each individual plant was labeled. Wheat grains at 10, 20, and 30 days after flowering were sampled, and three independent biological replicates were taken from each sample. The collected samples were immediately frozen in liquid nitrogen and then stored at -80 °C for later experimental use.

### 3.2. RNA Isolation and Sequencing

Total RNA from seeds was isolated using mirVana^TM^ miRNA ISOlation Kit (Thermo Fisher Scientific, Shanghai, China) and quantified using a Nano Drop 2000 spectrophotometer. Ribosomal RNA was digested using TruSeq Stranded Total RNA LT—(with Ribo Zero Plant) kit. The RNA was broken into short fragments by adding interruption reagents. A single strand of cDNA was synthesized using a six-base random primer. dUTP was used instead of dTTP during cDNA double strand synthesis, and different splices were connected. Then, a single strand containing dUTP was digested using UNG enzyme, leaving only one strand of cDNA connecting different splices of the strand. The purified cDNA strand underwent end repair, A-tail addition, and sequencing connections, followed by fragment size selection and PCR amplification. After passing the quality inspection of the constructed RNA library using Agilent 2100 Bioanalyzer, the Illumina sequencing machine was used for sequencing.

### 3.3. Quantitative and Differential Expression Analysis of Protein-Coding Genes

To quantify and analyze the differential expression of protein-coding genes, CleanReads were aligned to a specified reference genome using HISAT2, which provided positional information on the reference genome or genes, as well as unique sequence characteristics of the sequencing samples [49]. The database, comprising known reference gene sequences and annotation files, was utilized to determine the expression abundance of each protein-coding gene across samples through sequence similarity comparisons. The HTSeq-counts (version 0.9.1) software was employed to quantify the number of reads mapped to protein-coding genes in each sample, while the expression levels [50], in terms of fragments per kilobase of transcript per million mapped reads (FPKM) values, were calculated using Cufflinks (version 2.2.1) software. The normalization of mRNA counts within each sample was performed using DESeq (version 1.18.0) software (employing BaseMean values to estimate expression levels) [51,52], followed by the calculation of fold changes in expression. The significance of read count differences was tested using a negative binomial distribution test (NB). Finally, differentially expressed mRNAs were selected based on fold change and the results of significance tests.

### 3.4. Identification and Differential Expression Analysis of Non-Coding lncRNA Genes and circRNAs

Based on the genomic alignment results of each sample, transcripts were reassembled using the StringTie2 (version 1.3.3b) software. The merged transcripts were then compared individually to reference transcripts using the Cuffcompare (version 2.2.1) software, filtering out the new transcripts of known coding transcripts or known loci. Non-coding transcripts were selected based on the criteria of being longer than 200 nucleotides and having two or more exons. To predict the coding potential, four software tools (CPC2 (version beta), CNCI (version 1), Pfam (version v30), and PLEK (version 1.2)) were utilized, and transcripts with potential coding abilities were excluded to obtain a set of non-coding lncRNA transcripts [53,54,55,56].

For the prediction of circRNAs, the find_circ tool was used. Sequencing reads were aligned to the reference genome [36], and reads with continuous linear alignments were discarded, retaining only those reads with non-continuous linear alignments. Portions of the sequences from the 5′ and 3′ ends of these reads (5′ and 3′ anchors) were then realigned to the reference genome. Anchor positions arranged in reverse orientation were selected, and the sequences between the extended alignments were examined, with splicing sites conforming to the GT/AG rule. Finally, circRNAs and related annotation information were outputted.

The normalization of the counts of lncRNA genes and circRNAs in each sample was performed using the DESeq software (employing BaseMean values to estimate expression levels), followed by the calculation of fold changes in expression. The significance of read count differences was tested using a negative binomial distribution test (NB). In the end, differentially expressed lncRNA genes and circRNAs were selected based on fold change and the results of significance tests.

### 3.5. Enrichment Analysis of Differential mRNA, lncRNA Neighboring Genes, and circRNA Host Genes in GO and KEGG Pathways

For the enrichment analysis of differentially expressed protein-coding genes, all protein-coding genes were used as the background list, and differentially expressed protein-coding genes were selected as the candidate list from this background. The significance of GOterm enrichment within the candidate list was calculated using the hypergeometric distribution test to obtain *p*-values [57], which were then adjusted for multiple testing corrections using the Benjamini and Hochberg method to derive q-values. Pathway analysis for these genes was conducted using the KEGG database (combined with KEGG annotation results), and the significance of enrichment in each pathway was calculated using the hypergeometric test [58].

For the neighboring genes of differentially expressed lncRNA genes, GO enrichment analysis was performed to describe their functions (in conjunction with GO annotation results). All neighboring genes of lncRNA genes served as the background list, with the list of the neighboring genes of differentially expressed lncRNA genes acting as the candidate list. The hypergeometric test was employed to calculate the *p*-values for the significance of GO term enrichment within this list, and these *p*-values were adjusted using the Benjamini and Hochberg method to obtain q-values. For KEGG enrichment analysis, the KEGG database was used to perform pathway analysis on the neighboring genes of differentially expressed lncRNA genes (combined with KEGG annotation results), and the hypergeometric test was applied to determine the significance of enrichment within each pathway.

To identify the host genes of circRNAs, the annotation information of protein-coding genes and transcripts released in the database was used. By comparing location information, transcripts with the maximum overlap with circRNAs were determined to establish host genes. All circRNA host genes were used as the background list, with the list of differentially expressed circRNA host genes serving as the candidate list. The hypergeometric test was utilized to calculate the *p*-values for the significance of the GO term enrichment within this list, which were then adjusted using the Benjamini and Hochberg method to derive q-values. For KEGG pathway analysis, the KEGG database was used to analyze the differentially expressed circRNA host genes (combined with KEGG annotation results), and the hypergeometric test was used to calculate the significance of enrichment for each pathway entry.

### 3.6. Prediction of lncRNA and circRNA Target Relationships and Network Construction

Given that lncRNA genes and circRNAs contain multiple miRNA binding sites, methods used for predicting miRNA target genes can be employed to identify lncRNA genes and circRNAs that bind with miRNAs. The functional annotations of the miRNA target genes can then be used to elucidate the functions of these lncRNA genes and circRNAs. For plants, the TargetFinder (version 1.2) software is utilized for prediction purposes. For the overall enrichment results of differentially expressed lncRNA genes and circRNAs, the interactions are sorted by *p*-value. The top 300 miRNA–lncRNA and miRNA–circRNA interaction pairs with the lowest *p*-values are extracted. Using the R package ‘network’, an interaction network diagram of lncRNA–miRNA and circRNA–miRNA targets is constructed.

### 3.7. Quantitative Validation by qRT-PCR

For miRNAs, quantitative validation was performed using qRT-PCR with a tailing approach. Total RNA was extracted from six samples (materials from AnNong 0942 and AnKe 2005 at 10, 20, and 30 days) using TRIzol reagent. Reverse transcription was carried out using the SPARKScript miRNA 1st Strand cDNA Synthesis Kit (Tailing Reaction) (Qingdao, China). For target genes, cDNA was synthesized using the SPARKscript II RT Plus Kit (with gDNA Eraser). Quantitative real-time PCR was conducted on a Bio-Rad CFX-96 Touch system (Bio-Rad Laboratories, Hercules, CA, USA). Wheat actin was employed as the reference gene. All reactions were run in triplicate, and relative expression levels were calculated using the 2^^−ΔCT^ method.

### 3.8. Double Luciferase Experiment

The expression vector pCAMBIA1300 and the dual luciferase expression vector pGreenII 0800-LUC were preserved in our laboratory, and the pri miRNA sequence was constructed by the biosynthesis of Cyprinaceae. Based on the sequence of the target gene TaTGW-7B, by analyzing the possible binding sites with tae-miR1130a (5′-CUCCGUCUCUGUAAUGUAAGACG-3′), 190 bp containing all binding sites were selected for gene synthesis and inserted into the pGreenII 0800-LUC vector to construct the wild-type WT-TaTGW-7B-pGreenII 0800-LUC plasmid and the MUT-TaTGW-7B-pGreenII 0800-LUC plasmid with binding site mutations. Similarly, based on the TraesCS6D02G268200 sequence, we analyzed the possible binding sites with tae-miR1122a (5′-UGAUACAUCGUAGA-3′), and selected 122 bp containing all binding sites for gene synthesis, and inserted these into the pGreenII 0800-LUC vector to construct the wild-type WT-TaNF-YB1-D-pGreenII 0800-LUC plasmid and MUT-TaNF-YB1-D-pGreenII 0800-LUC plasmid with binding site mutations. DNA sequencing on the synthesized plasmid was performed. The sequencing results were compared with the TaTGW-7B (TraesCS7B02G131900) and TaNF-YB1-D (TraesCS6D02G268200) sequences in NCBI, and obtained a 100% alignment rate of plasmid retention bacterial fluid.

The plasmids were introduced into Agrobacterium GV3101 and coated on LB solid culture medium (kanamycin 50) μG/mL + gentamicin 50 μG/mL). PCR was used to screen for positive transformants. Single-colony shaking bacteria were picked from the plate, cultured at 28 °C overnight, and then transferred to new culture medium according to the ratio of 1:1000, 5000 r/5 min, 22 °C, centrifugation for 5 min to collect bacteria. The suspension of bacteria was carried out in a transformation buffer containing 10 mmol/L MgCl_2_, 10 mmol/LMES, 150 μmol/L acetosyringone, and at pH5.7, and the final concentration was OD600 = 1.0. After standing at room temperature for 3 h, the two agrobacteria were mixed according to the volume of 1:1 and injected into tobacco leaves. LUC/REN value was measured.

## 4. Discussion

Currently, wheat yield has achieved a significant improvement, but there are still some inherent limitations, among which increasing the yield remains one of the significant challenges in the breeding program. The wheat yield is mainly influenced by the comprehensive effects of panicles per unit area, grains per panicle, and 1000-grain weight. This is especially the case for the 1000-grain weight, which is a key factor affecting wheat yield. So far, researchers have successfully cloned many genes related to 1000-grain weight [59]. However, the role of lncRNA and circRNA in wheat grain development is unclear. Two wheat varieties with significant differences in 1000-grain weight were used to conduct full transcriptome analysis of their grains at different time points 10, 20, and 30 days after flowering. The aim was to investigate the effects of lncRNA and circRNA on wheat grain development.

The differences in lncRNA and circRNA expression that affect the 1000-grain weight of wheat were identified. The functional enrichment analysis of differentially expressed genes showed that these non-coding RNA target genes were mainly concentrated in biological processes such as photosynthesis, carbohydrate metabolism, cellular component organization, starch binding, and nutrient pool activity during wheat grain development. The processes of photosynthesis, carbon metabolism, and nutrient pool activity are closely related to energy storage and metabolism. Nutrient pool activity covers the adsorption, desorption, transformation, and release of nutrients in soil, as well as the ability of microorganisms to grow, metabolize, and decompose organic matter, which is extremely important for soil fertility and plant growth. By maintaining appropriate nutrient pool activity, the soil can provide the necessary nutrients for plants, which is crucial for their growth and development. At the same time, photosynthesis provides the main energy and carbon source for wheat grains, which has a significant impact on the growth and development of wheat grains. Chi et al. [60] pointed out that many genes are involved in the organization of cellular components, biogenesis, and nutrient pool activity during wheat grain development. The study by Niu et al. [61] showed that prolonging the photosynthesis cycle would increase the grain weight of wheat. Luo et al. [62] successfully achieved an increase in the 1000-grain weight of wheat by improving the carbohydrate metabolism activity of high-quality grains. Our study suggests that photosynthesis, carbohydrate metabolism activity, and nutrient pool activity are closely related to the increase in the 1000-grain weight of wheat. The possible mechanism is that the differential expression of lncRNA and circRNA enhances the photosynthesis intensity and carbon metabolism function, thereby enhancing the photosynthetic capacity and nutrient transport speed, increasing the accumulation of photosynthetic products, providing abundant nutrients for wheat grains, and ultimately leading to an increase in wheat grain weight per thousand grains. This study provides a useful reference for further research on the functions and genes related to the 1000-grain weight of wheat.

With the continuous progress of transcriptome sequencing technology, multiple genes related to 1000-grain weight in wheat have been revealed. For example, TaGW2 and TaDA1 have an additive effect on the 1000-grain weight of wheat, TaGW2 has a negative regulatory effect on the heat grain weight [59]. The knockout expression of the TaPIN1 gene can increase the tillering number of transgenic wheat lines [63]. MiRNA has also been found to play a crucial role in indirectly regulating grain development. For example, miRNA (PC-5p-2614_215) plays a crucial role in grain development and nitrogen response, which determines the weight and quality of wheat grain. miR396 has an effect on wheat grain development by regulating the expression of GRF genes [64]. In this study, two wheat grains with significant differences in their 1000-grain weight were analyzed by full transcriptome sequencing technology. A variety of lncRNA, circRNA, and miRNA interacting with IncRNA and circRNA were identified. The interaction mechanism of LncRNA–miRNA and circRNA–miRNA plays an important role in the growth and development of plants. The specific mechanisms by which lncRNA plays a regulatory role in miRNA are as follows: lncRNA indirectly inhibits the negative regulation of miRNA on target genes by competitively binding to 3′-UTR of miRNA target mRNA; as a competitive and endogenous RNA, lncRNA acts as a “molecular sponge” of miRNA and inhibits its expression; as a potential pri-miRNA, mature miRNA is produced, which indirectly regulates the expression of target motifs. However, circRNAs are rich in the binding sites of miRNA, and by binding with specific miRNA, the inhibition of miRNA on its target gene can be relieved. Our analysis of the interaction between lncRNA–miRNA and circRNA–miRNA target networks shows that TCONS_00070295-miR5175-5p, TCONS_00070295-miR1133, tcons_00070299-miR1127a, TCONS_00066149-miRNA1122a, circRNA_0587-miR1130a, circRNA_1891-miR1117, and circRNA_0917-miRNA1133 are widely existed in the development of wheat grains. We used psRobot to predict the target of miRNA, and based on the annotation results, we found that there were two cloned genes TaNF-YB1-D (TRAESCS 6D02G2068200) and TaTGW-7B (TRAESCS 7B0358400) in the downstream of target gene. The quantitative analysis of these two genes and their upstream miRNA (miR1122a and miR1130a) by qRT-PCR showed that the expression of TaNF-YB1-D and TaTGW-7B in the grain of Annong 0942 developed for 20 d was higher than that in Anke 2005. However, the expression of their corresponding upstream miRNA in the grain of Annong 0942 developed for 20 d was lower than that in Anke 2005. This indicates that there is a potential targeting relationship between these two important grain weight genes and their miRNA. In order to further verify this hypothesis, we conducted an analysis of the interactions within the lncRNA–miRNA and circRNA–miRNA targeting networks. The results revealed that circRNAs participate in more complex and extensive interaction networks during seed development and grain size formation, with tae-miR1177, tae-miR1128, and tae-miR1130b-3p serving as central hubs, This indicates that there may be multiple circRNAs co-regulating the same miRNA, which is not uncommon in wheat. Kumar’s research on wheat showed that the TaSPX gene was targeted by nine different miRNAs [65]. In contrast, lncRNA genes formed a singular network centered on tae-miR1133 and tae-miR5175-5p only in the inter-variety comparisons. Using psRobot, we predicted the target genes of miRNAs and identified two downstream clonal genes, TaNF-YB1-D (TraesCS 6D02G2068200) and TaTGW-7B (TraesCS02G7B0358400), based on the annotation results. The quantitative analysis of these two genes and their upstream miRNAs (miR1122a and miR1130a) through qRT-PCR showed that the expression of TaNF-YB1-D and TaTGW-7B was higher in the spikelets of Annong 0942 at 20 days of development compared to Anke 2005. However, the expression of the corresponding upstream miRNAs was lower in the spikelets of Annong 0942 at 20 days of development than in Anke 2005. This suggests a potential targeting relationship between these two important spikelet weight genes and their miRNAs. To further validate this hypothesis, we conducted a dual-luciferase reporter assay on these gene pairs (miR1122a and TaNF-YB1-D, miR1130a, and TaTGW-7B). The results confirmed the targeting relationship with a high expression of miR1130a and miR1122a leading to the cleavage of TaTGW-7B and TaNF-YB1-D, thereby affecting the variation in spikelet weight. Other studies have also shown that TaTGW-7B can regulate the 1000-grain weight during wheat spikelet development, and the knockout of the TaNF-YB1-D gene leads to the abnormal development of wheat spikelets [3,16]. Interestingly, the single nucleotide polymorphism of the Q gene, the earliest cloned domestication gene in wheat, weakened the affinity between miR172 and its binding. The weakening of this affinity leads to an increase in Q gene expression, which affects the morphology of wheat spikes and the threshing ability of grains [7]. Correspondingly, we found the presence of miR1130a and miR1122a in varieties with grain weight differences, where miR1130a targets a homologous copy of the candidate gene TaTGW-7A for quantitative trait loci related to grain weight [3], suggesting a possibility that miRNA and its target genes often co-evolve under selection pressure during long-term evolution. With the transition of wheat from wild to cultivated varieties, the demand for more effective agronomic traits has driven the evolution of the interaction between non-coding RNA and target genes to meet human agricultural needs. This multifactorial regulation may also play a role in controlling complex quantitative trait loci such as grain weight, which is influenced by multiple genes and environmental factors, and may involve multiple miRNAs and their corresponding target genes. The interaction between these genes and miRNAs forms a complex regulatory network. We further found that the expression levels of circRNA-0957, circRNA_1041, circRNA-0587, and circRNA_2127 in Anong 0942 were higher than those in Anke 2005. Thus, we hypothesize that circRNA_0957, circRNA_1041, circRNA_0587, and circRNA_2127 may regulate the development of wheat spikelets in Annong 0942 through an interaction mechanism with miRNAs.

Comprehensive analysis shows that the 1000-grain weight of wheat is influenced by various factors, including gene expression, photosynthesis, carbohydrate metabolism, and nutrient pool activity. Although there is currently limited research on the expression and function of lncRNA and circRNA, as well as their interaction mechanisms, during wheat grain development, this study screened a portion of lncRNA and circRNA genes and potential interaction mechanisms that may affect the wheat grain weight through full transcriptome analysis, providing some data support for subsequent research. In future work, further functional verification of these genes and functions can be carried out to provide a more solid theoretical basis for understanding the molecular mechanism underlying wheat grain weight.

## 5. Conclusions

**Figure 6:**
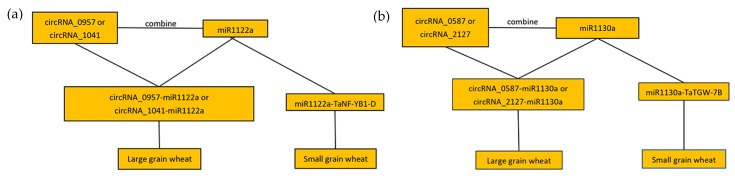
The regulatory pattern diagram of circRNA_0957 or circRNA_1041 and miR1122a (**a**) and the regulatory pattern diagram of circRNA_0587 or circRNA_2127 and miR1130a (**b**).

Although the yield of wheat has significantly increased, further increasing the yield remains a major challenge in breeding programs. The yield of wheat is influenced by the number of spikes per unit area, the number of grains per spike, and the 1000-grain weight, especially the 1000-grain weight, which plays a decisive role in the total yield. Full transcriptome analysis revealed key findings during wheat grain development: the differential expression of miR1130a and miR1122a resulted in significant changes in the transcripts of important grain development genes TaTGW-7B and TaNF-YB1-D. In addition, it is speculated that the upstream regulatory RNAs circRNA-0957 and circRNA_1041 may competitively bind with tae-miR1122a, thereby inhibiting the regulatory effect of miR1122a on TaNF-YB1-D. At the same time, circRNA:0587 and circRNA_2127 can specifically bind to miR1130a, relieving the negative regulatory effect on TaTGW-7B, thereby jointly affecting the grain weight of wheat (Figure 6). These new findings provide a new perspective and potential targets for understanding and regulating the molecular mechanism of wheat 1000-grain weight, opening up new directions for future wheat improvement research.

## Data Availability

Data are available upon request.

## Supplementary Materials

The supporting information can be downloaded at: https://www.mdpi.com/article/10.3390/ijms25105484/s1.
