# Supplementary material for: Identification and Analysis of lncRNA and circRNA Related to Wheat Grain Development
Source: Int J Mol Sci. 2024 May 17;25(10):5484. doi: 10.3390/ijms25105484 (PMC11122269; doi:10.3390/ijms25105484)
Supplement: Supplementary file 1 [file ijms-25-05484-s001.zip › ijms-2988347 supplementary figures.pdf]

Supplementary Materials

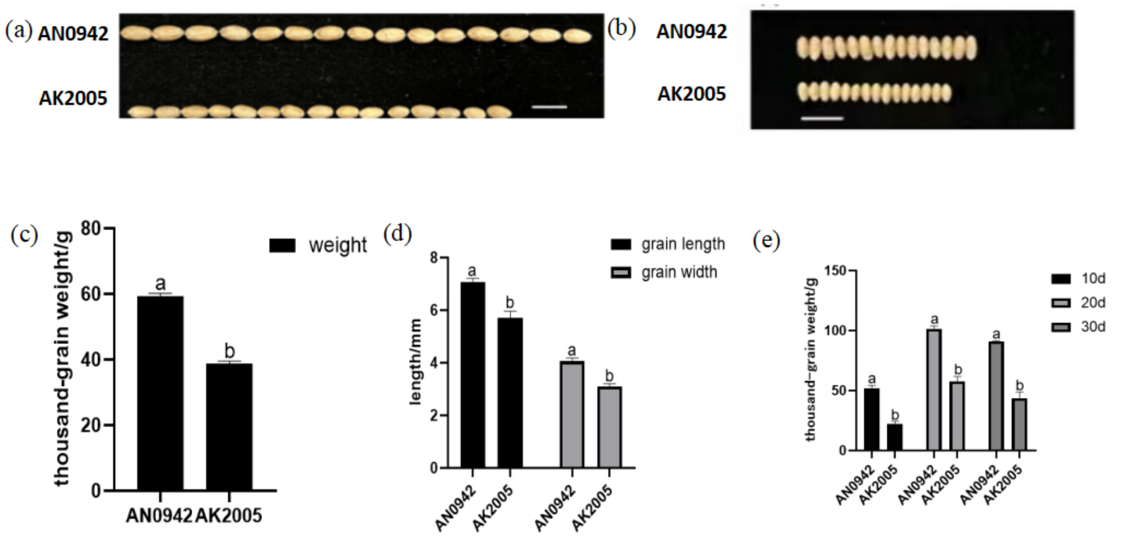

Figure S1. a and b showed the grain size of Annong 0942 and Anke 2005； c: the difference in thousand grain weight between Annong 0942 and Anke 2005; d: the difference in grain length and width between Annong 0942 and Anke 2005; e: the 1000-grain weight of wheat grains at 10d, 20d, and 30d after flowering in Annong 0942 and Anke 2005.

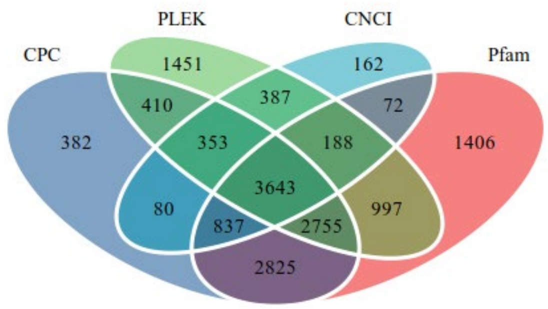

Figure S2. Wayne diagram of four software prediction results for candidate lncRNA encoding ability.

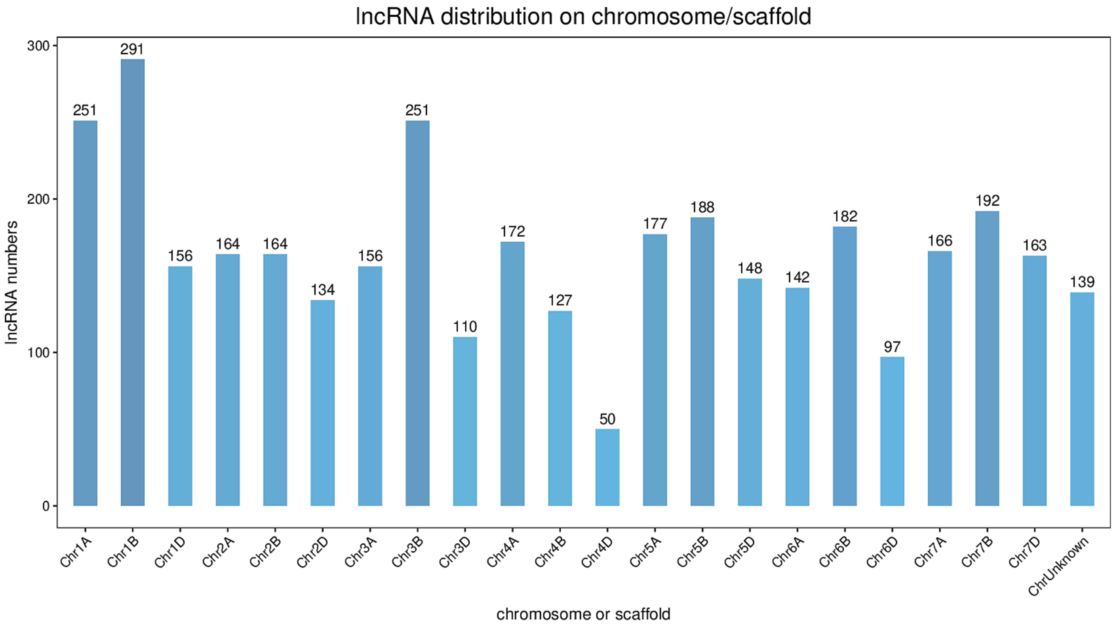

Figure S3. LncRNA distribution on chromosome or scaffold.

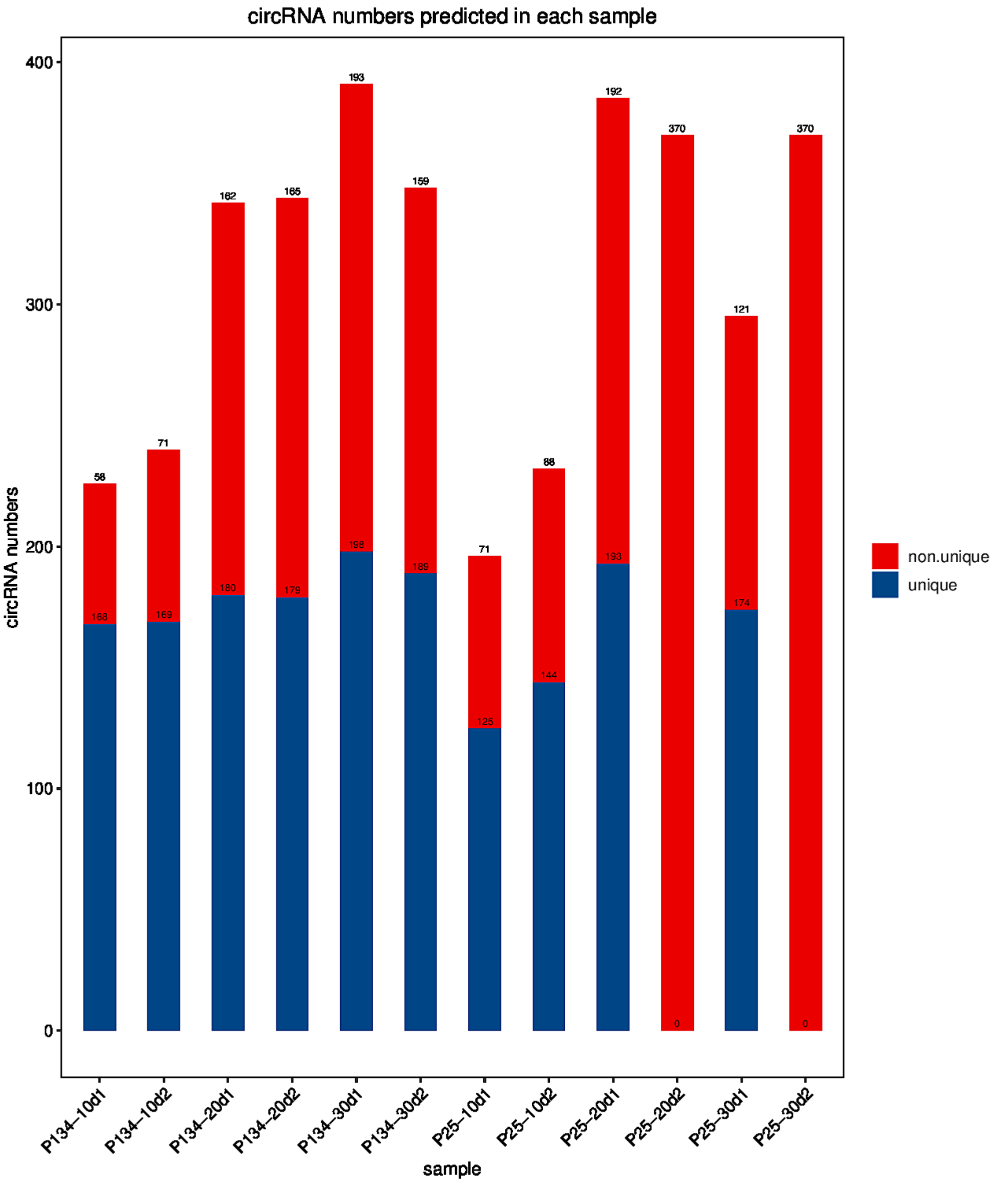

Figure S4. Statistical results of circRNA identified from different samples.

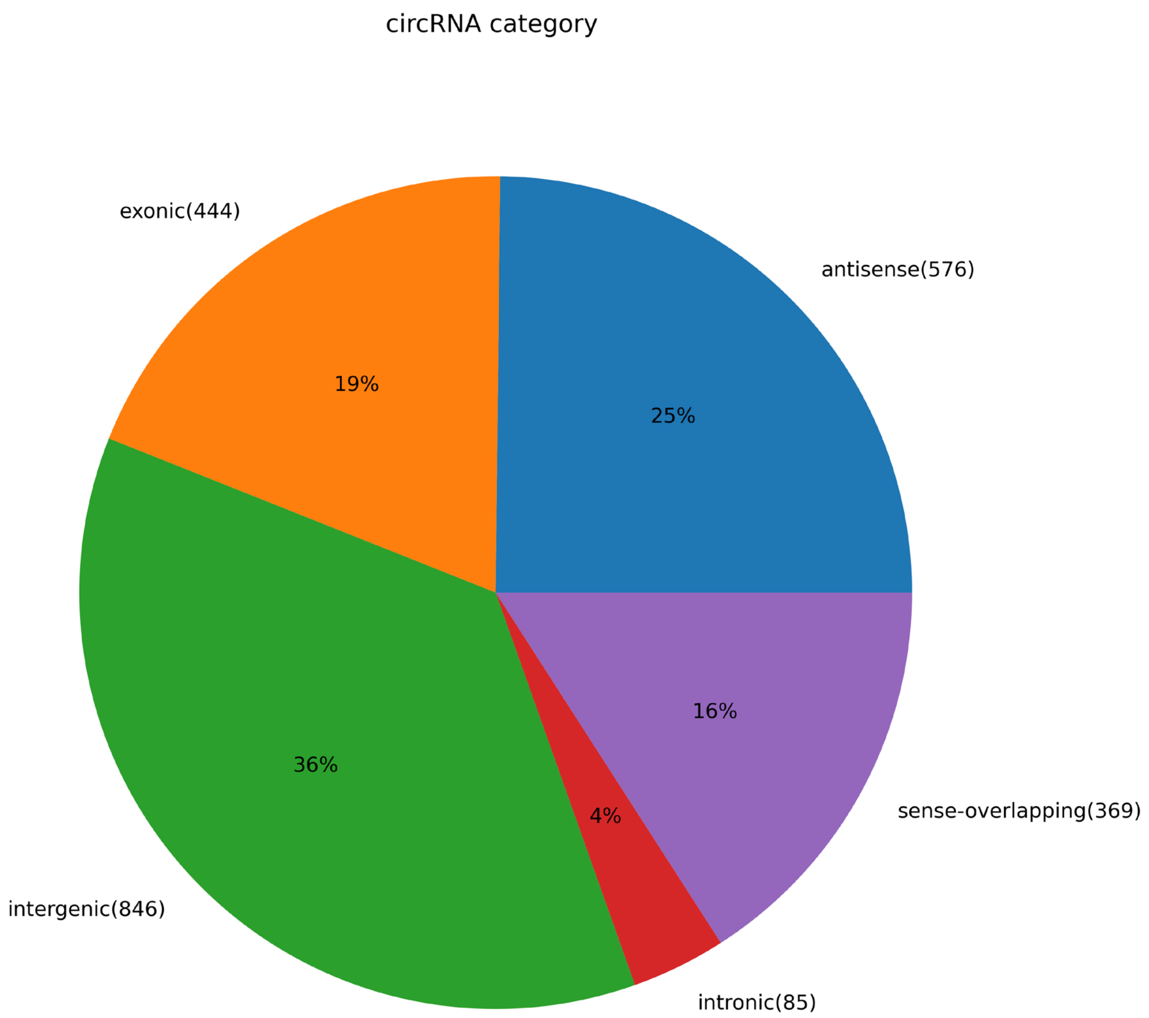

Figure S5. CircRNA type statistical chart.

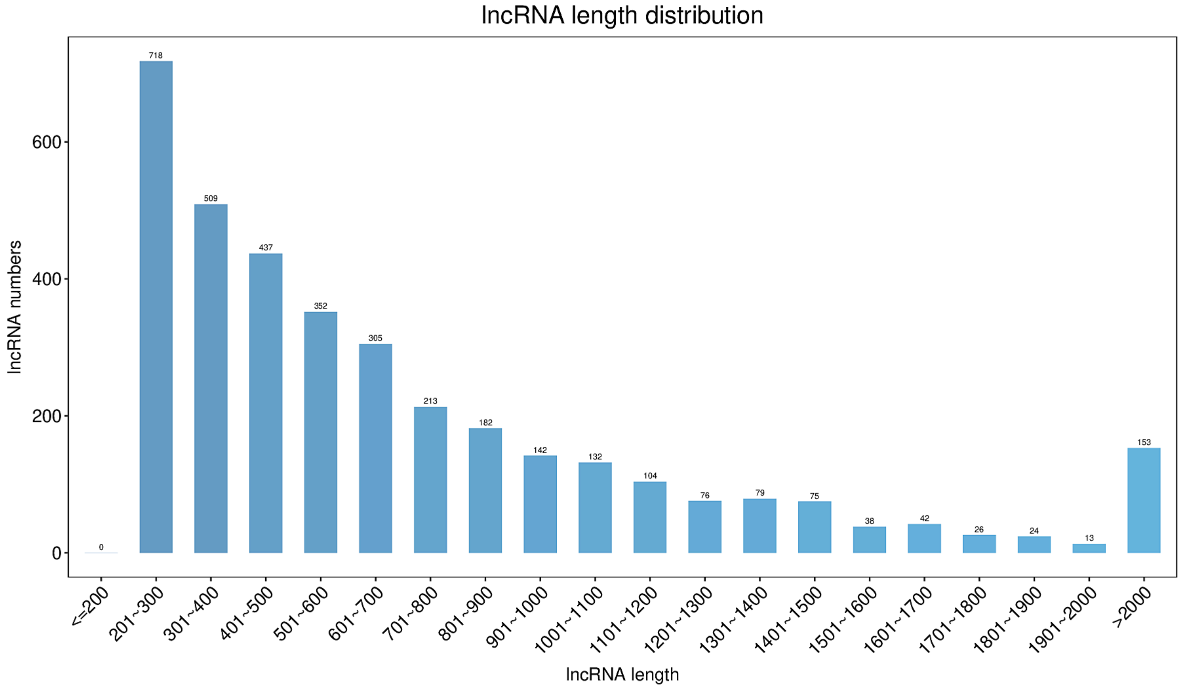

Figure S6. Distribution map of lncRNA sequence length.

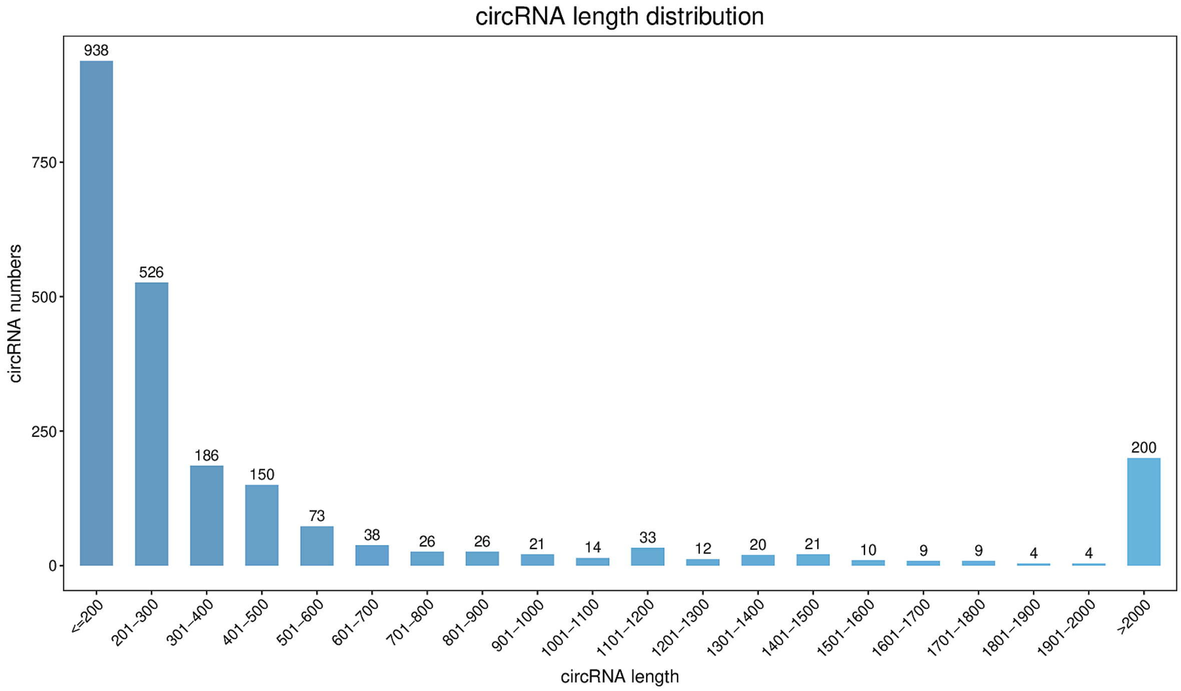

Figure S7. CircRNA sequence length distribution map.

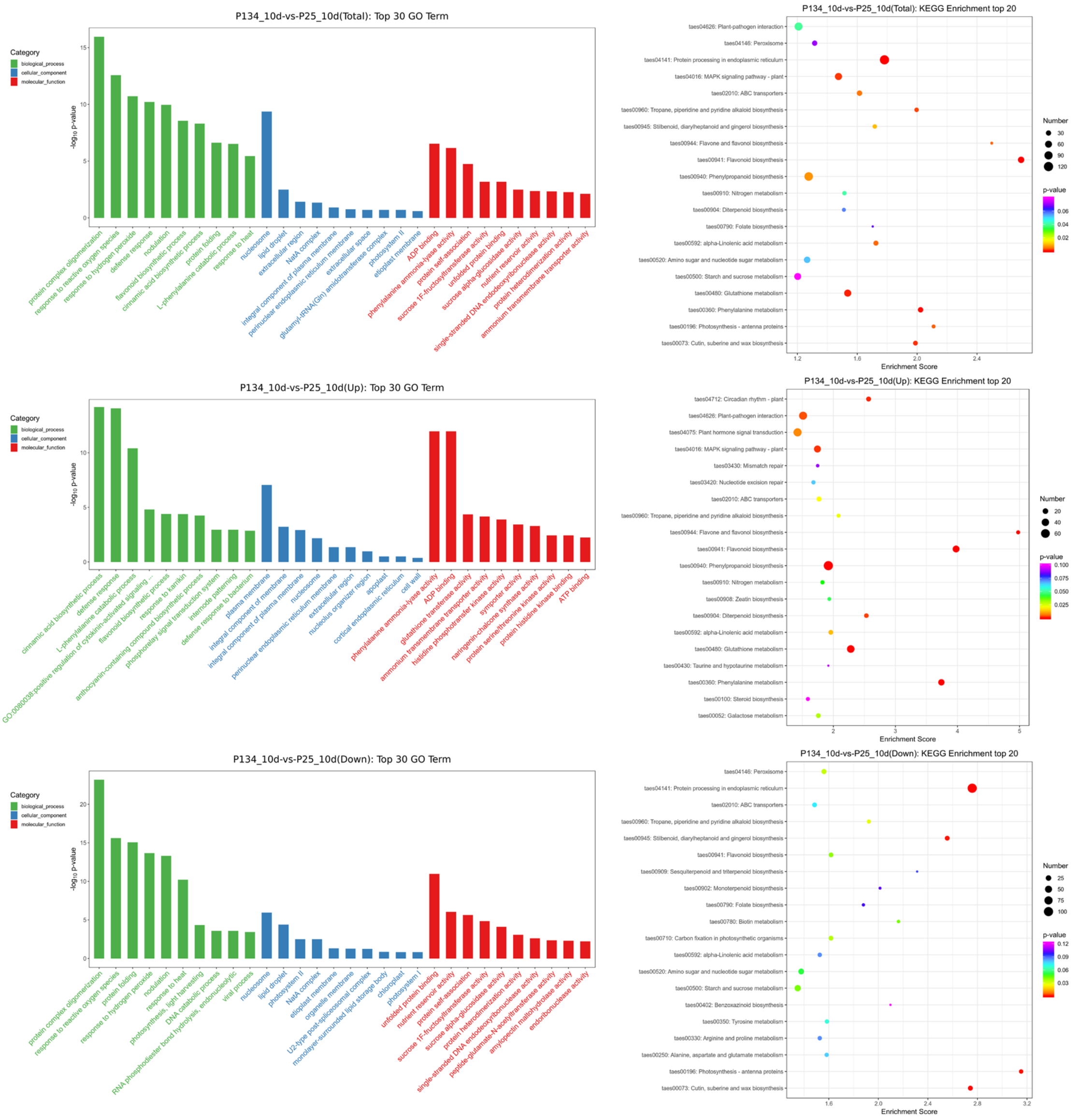

Figure S8. Enrichment Analysis of Differential mRNA GO and KEGG in Whole Transcriptome of Different Granules. (a) P134_ 10d vs P25_10d GO enrichment and KEGG enrichment analysis of all differentially expressed mRNA after 10 days; (b) P134_ 10d vs P25_ 10d differential expression upregulated mRNA GO enrichment and KEGG enrichment analysis; (c) P134_ 10d vs P25_ 10d differential expression downregulated mRNA GO enrichment and KEGG enrichment analysis.

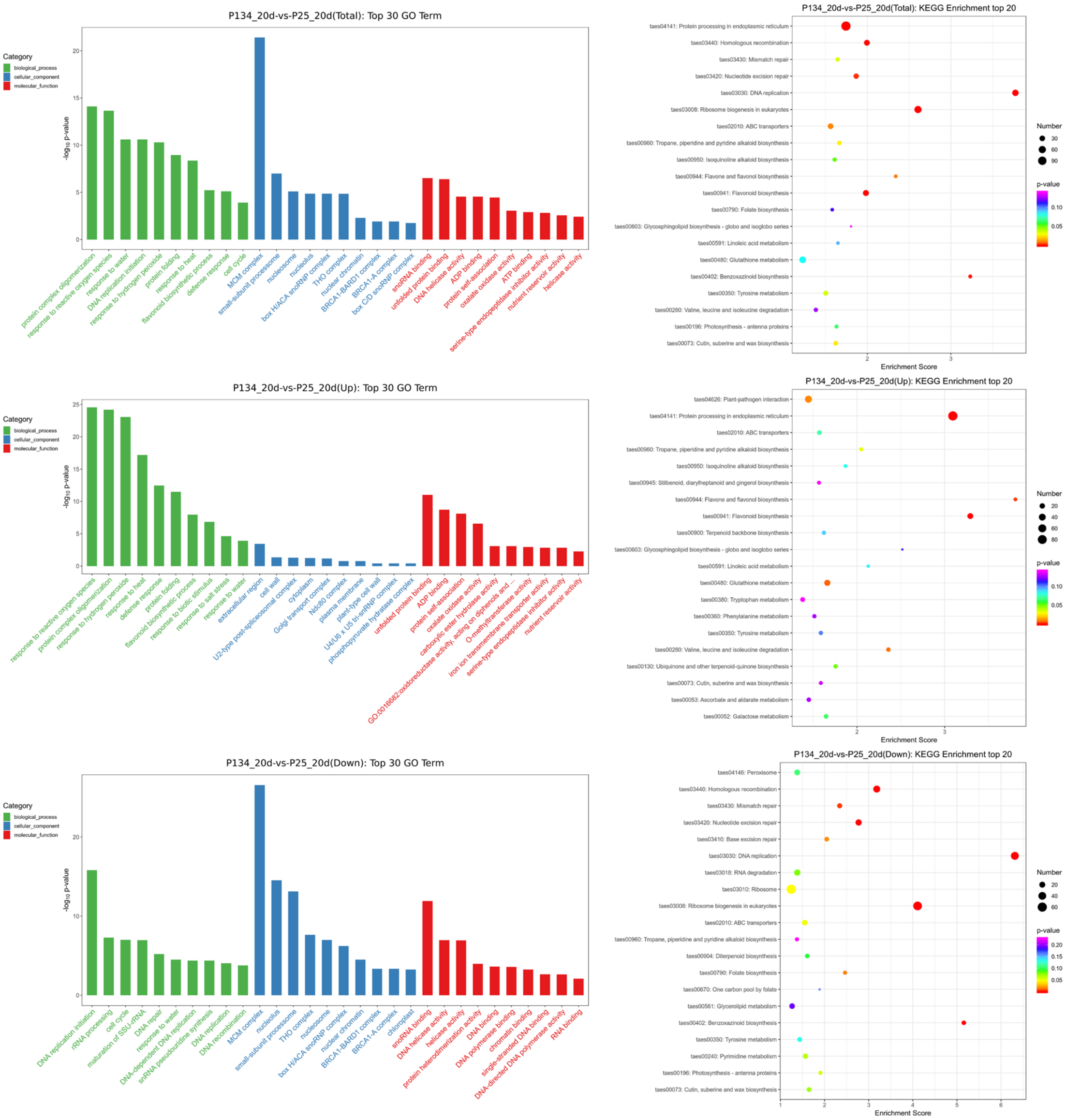

Figure S9. P134_ 20d vs P25_ 20d whole Transcriptome differential mRNA GO and KEGG enrichment analysis. (a) P134_ 20d vs P25_ 20d of differentially expressed mRNA GO enrichment and KEGG enrichment analysis; (b) P134_ 20d vs P25_ 20d differential expression upregulated mRNA GO enrichment and KEGG enrichment analysis; (c) P134_ 20d vs P25_ 20d differential expression downregulated mRNA GO enrichment and KEGG enrichment analysis.

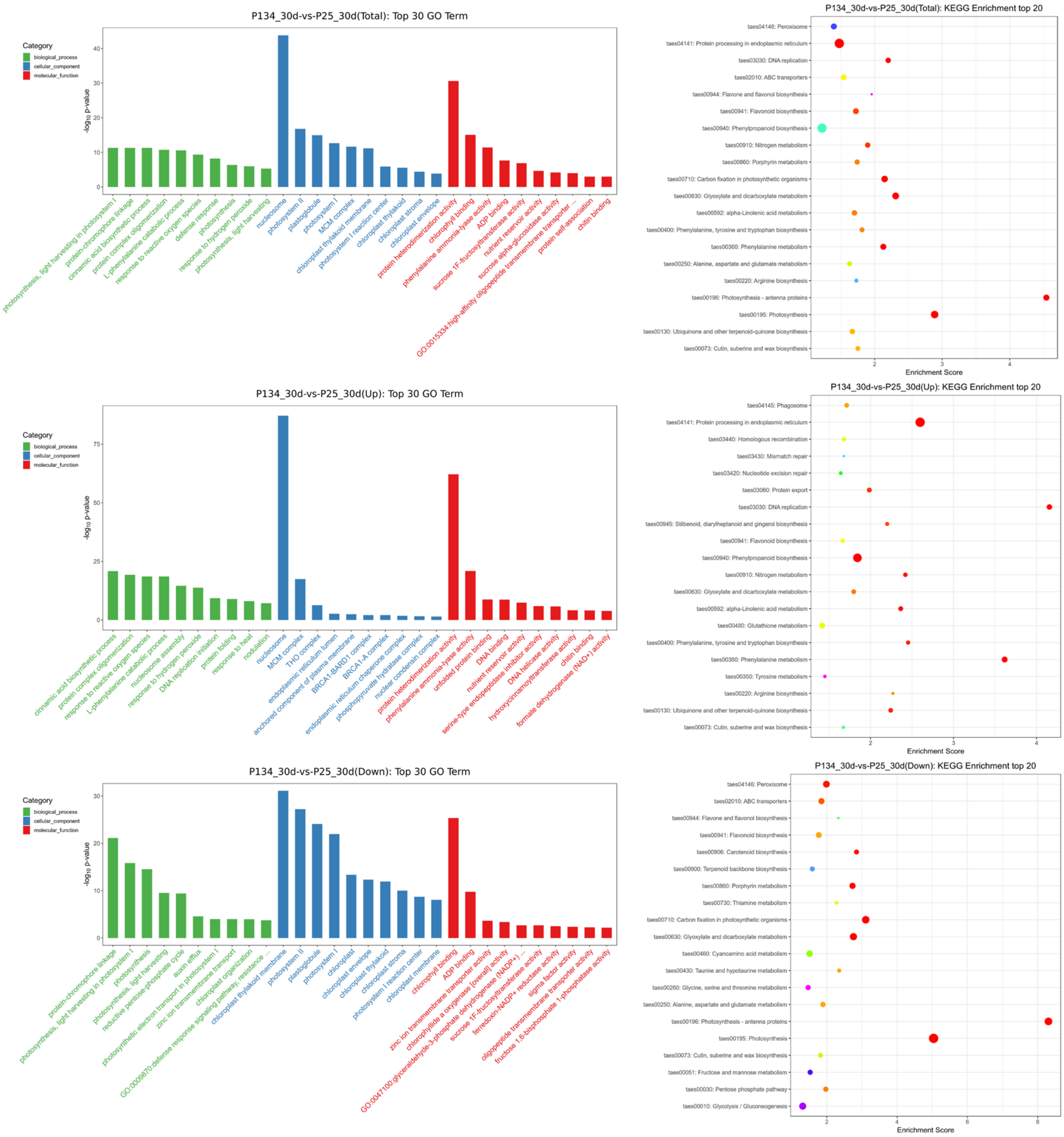

Figure S10. P134_ 30d vs P25_ 30-d whole Transcriptome differential mRNA GO and KEGG enrichment analysis. (aanalysis. (d vs P25_30d GO enrichment and KEGG enrichment analysis of all differentially expressed mRNA after 30 days; (b) P134_ 30d vs P25_ 30d differential expression upregulated mRNA GO enrichment and KEGG enrichment analysis; Differential expression downregulated mRNA GO enrichment and KEGG enrichment analysis. (c) P134_ 30d vs P25_ 30d differential expression downregulated mRNA GO enrichment and KEGG enrichment analysis.

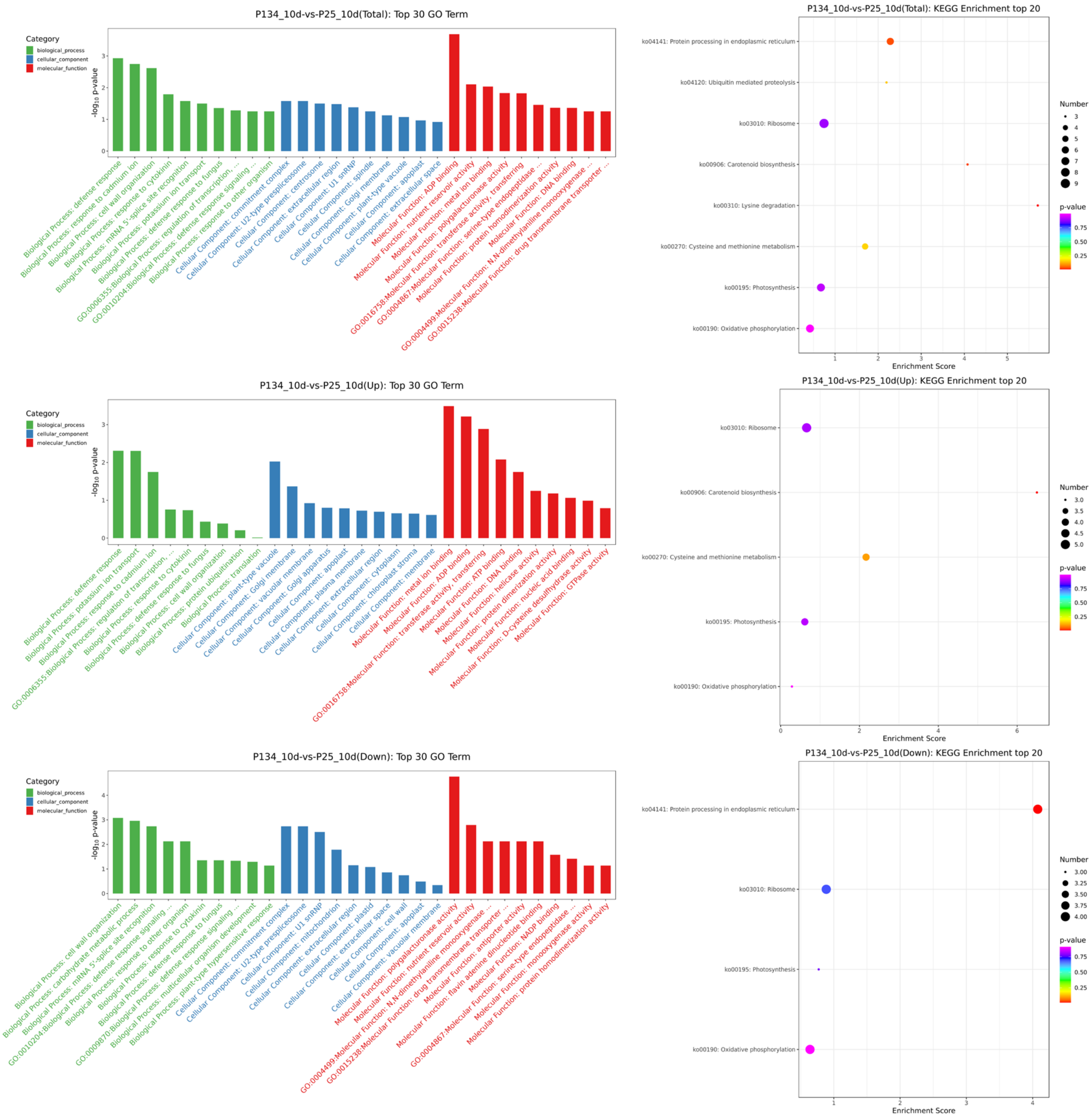

Figure S11. P134_ 10d vs P25_ 10d whole Transcriptome differential lncRNA GO and KEGG enrichment analysis. (analysis. (d vs P25_ 10d differential expression of lncRNA GO enrichment and KEGG enrichment analysis; (b) P134_ 10d vs P25_ 10d differential expression upregulated lncRNA GO enrichment and KEGG enrichment analysis; (c) P134_ 10d vs P25_ 10d differential expression downregulated lncRNA GO enrichment and KEGG enrichment analysis.

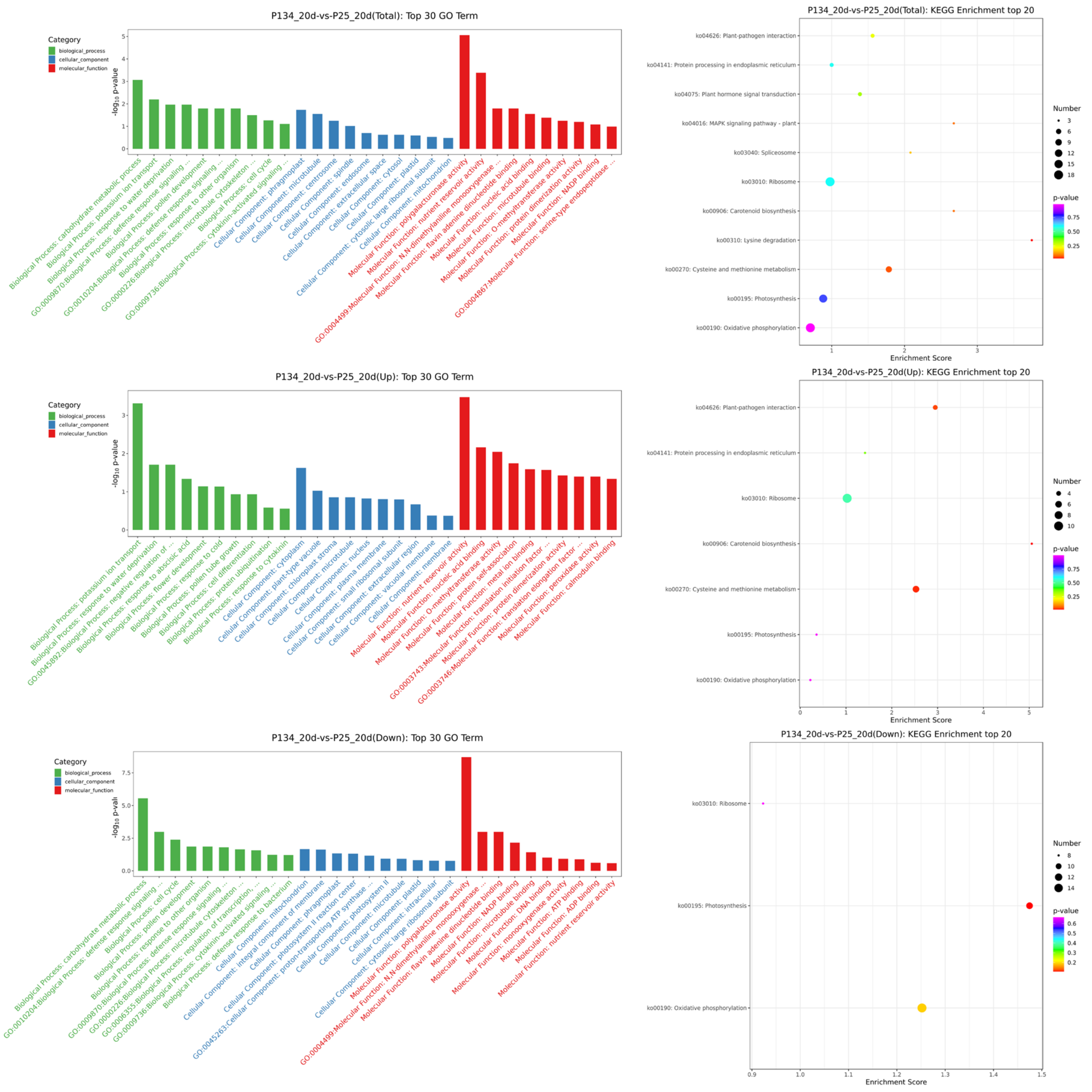

Figure S12. P134_ 20d vs P25_ 20d whole Transcriptome differential lncRNA GO and KEGG enrichment analysis. (a) P134_ 20d vs P25_ 20d differential expression of lncRNA GO enrichment and KEGG enrichment analysis; (b) P134_ 20d vs P25_ 20d differential expression upregulated lncRNA GO enrichment and KEGG enrichment analysis; (c) P134_ 20d vs P25_ 20d differential expression downregulated lncRNA GO enrichment and KEGG enrichment analysis.

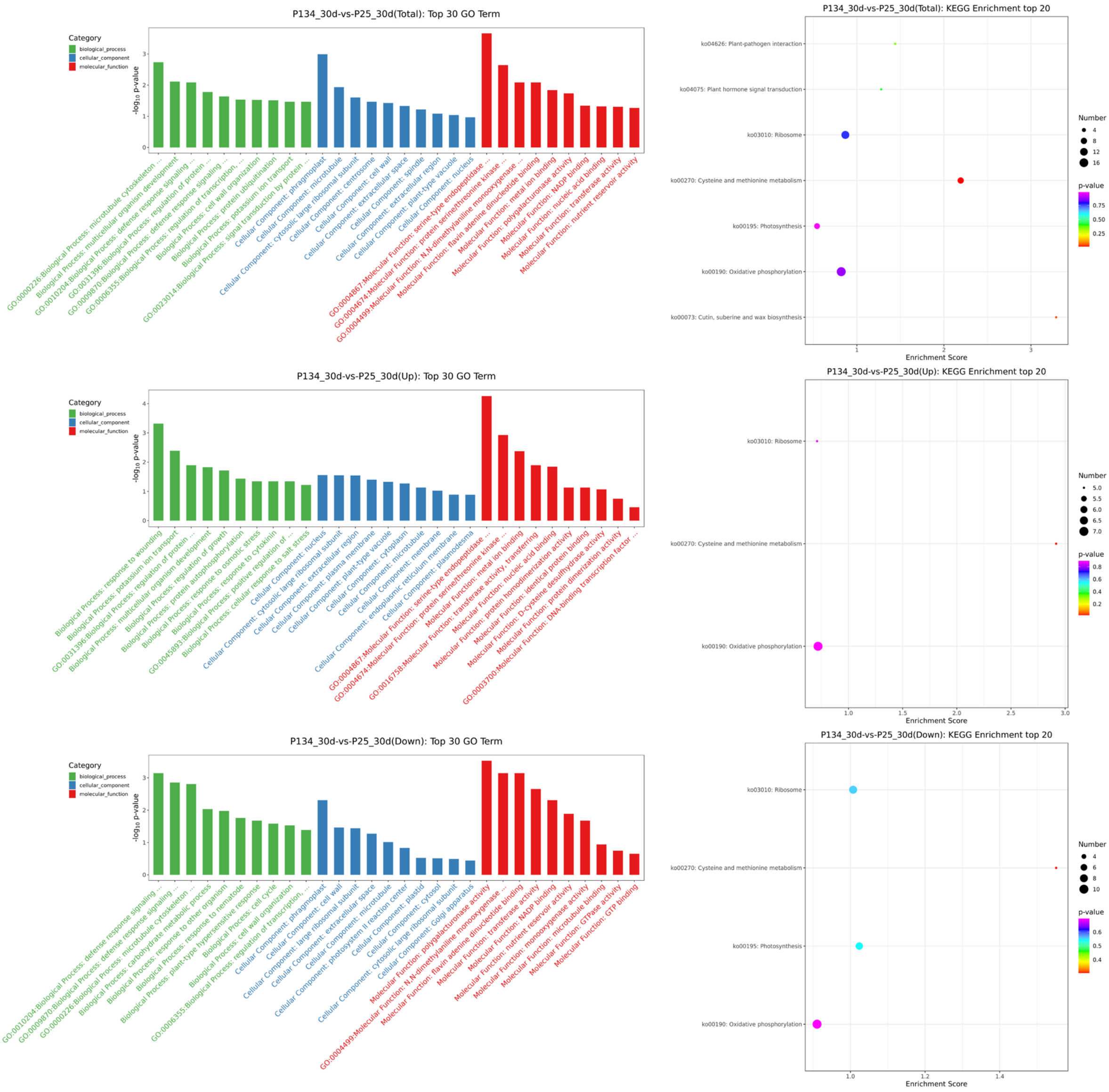

Figure S13. P134_ 30d vs P25_ 30d whole Transcriptome differential lncRNA GO and KEGG enrichment analysis. (a) P134_ 30d vs P25_ 30d differentially expressed lncRNA GO enrichment and KEGG enrichment analysis; (b) P134_ 30d vs P25_ 30d differential expression upregulated lncRNA GO enrichment and KEGG enrichment analysis; (c) P134_ 30d vs P25_ 30d differential expression downregulated lncRNA GO enrichment and KEGG enrichment analysis.

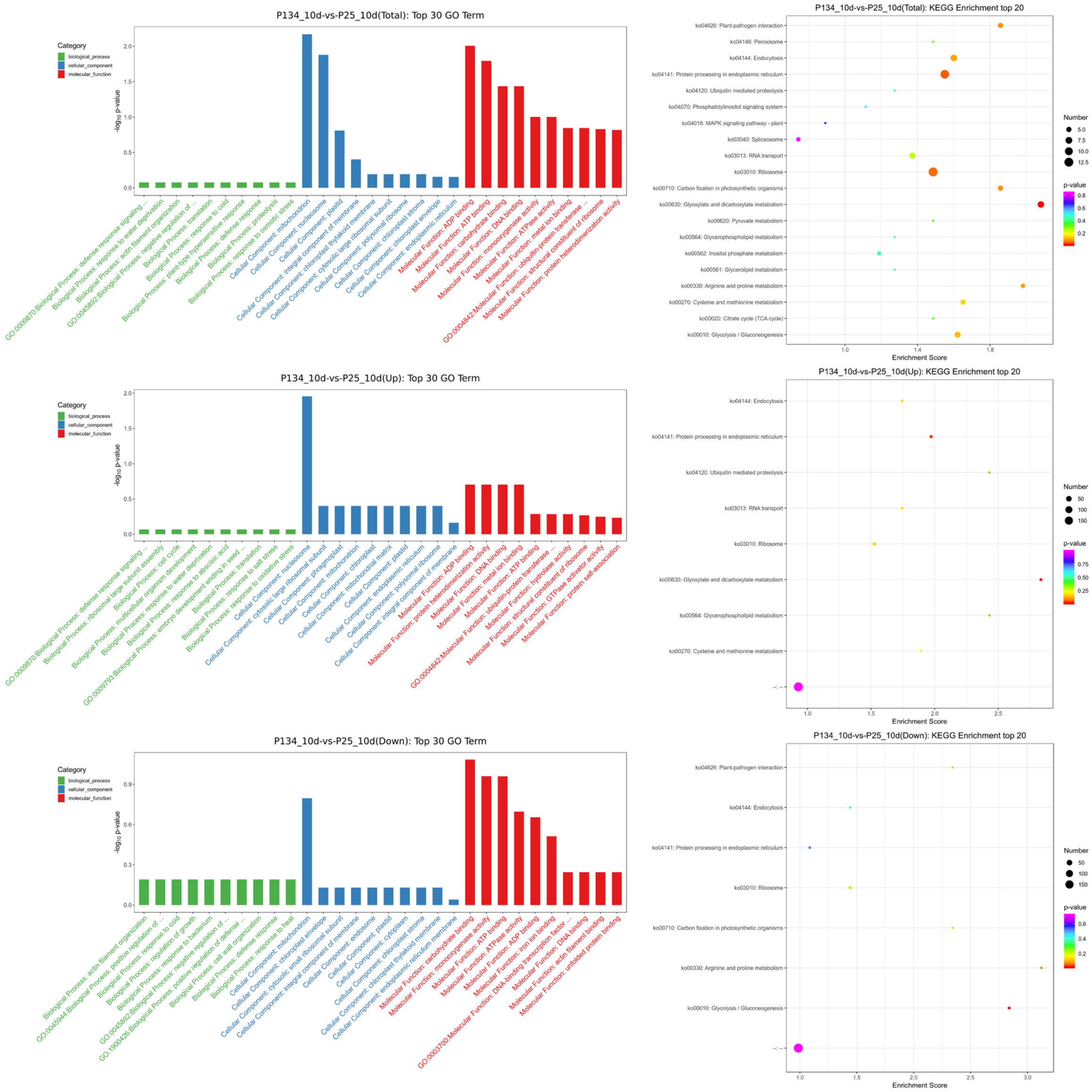

Figure S14. P134_ 10d vs P25_ 10d whole Transcriptome differential circRNA GO and KEGG enrichment analysis. (a) P134_ 10d vs P25_ 10d differential expression of circRNA GO enrichment and KEGG enrichment analysis; (b) P134_ 10d vs P25_ 10d differential expression upregulated circRNA GO enrichment and KEGG enrichment analysis; (c) P134_ 10d vsP25_ 10d differential expression downregulated circRNA GO enrichment and KEGG enrichment analysis.

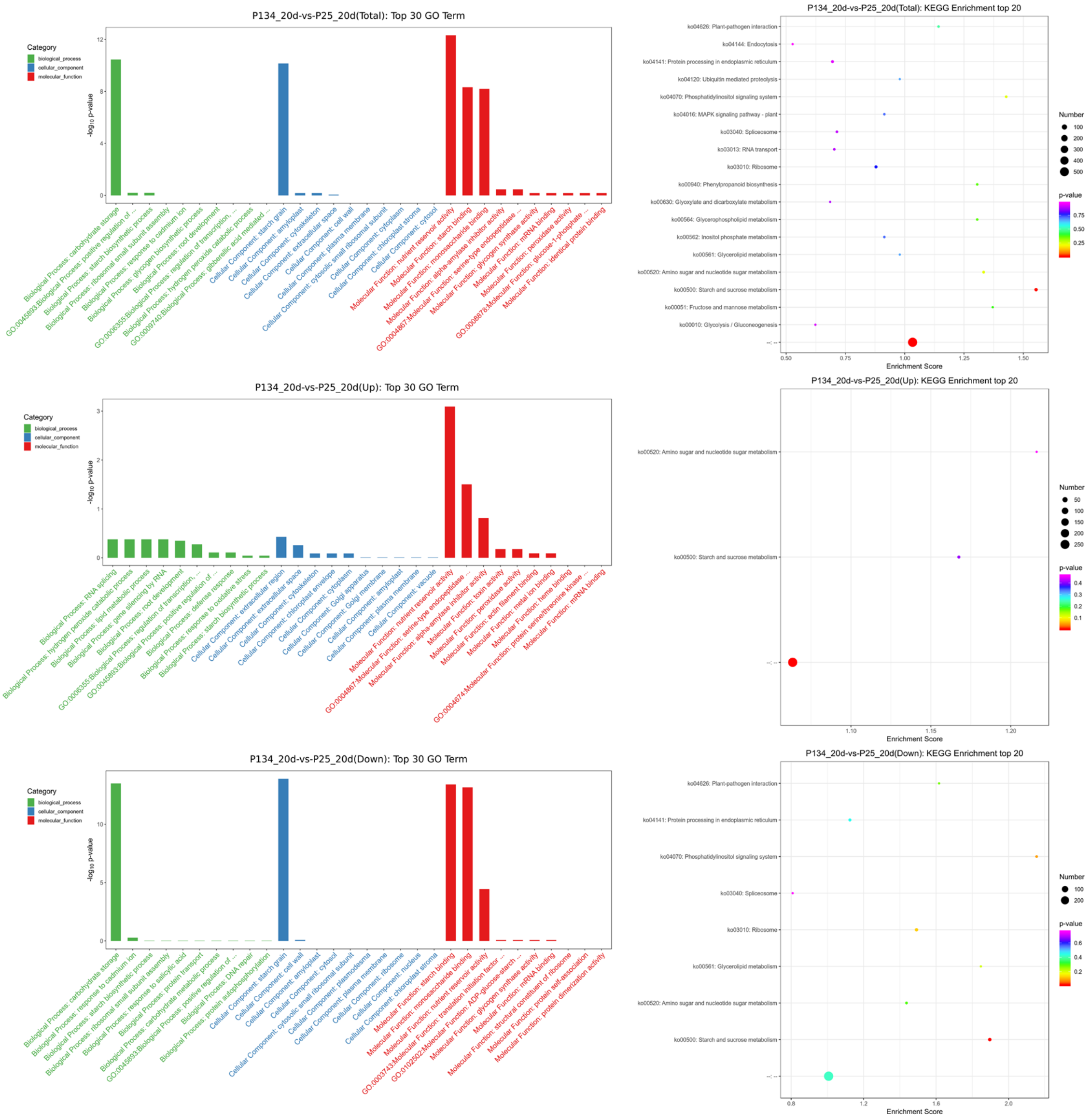

Figure S15. P134_ 20d vs P25_ 20d whole Transcriptome differential circRNA GO and KEGG enrichment analysis (a) P134_ 20d vs P25_ 20d differential expression of circRNA GO enrichment and KEGG enrichment analysis; (b) P134_ 20d vs P25_ 20d differential expression upregulated circRNA GO enrichment and KEGG enrichment analysis; (c) P134_ 20d vs P25_ 20d differential expression downregulated circRNA GO enrichment and KEGG enrichment analysis.

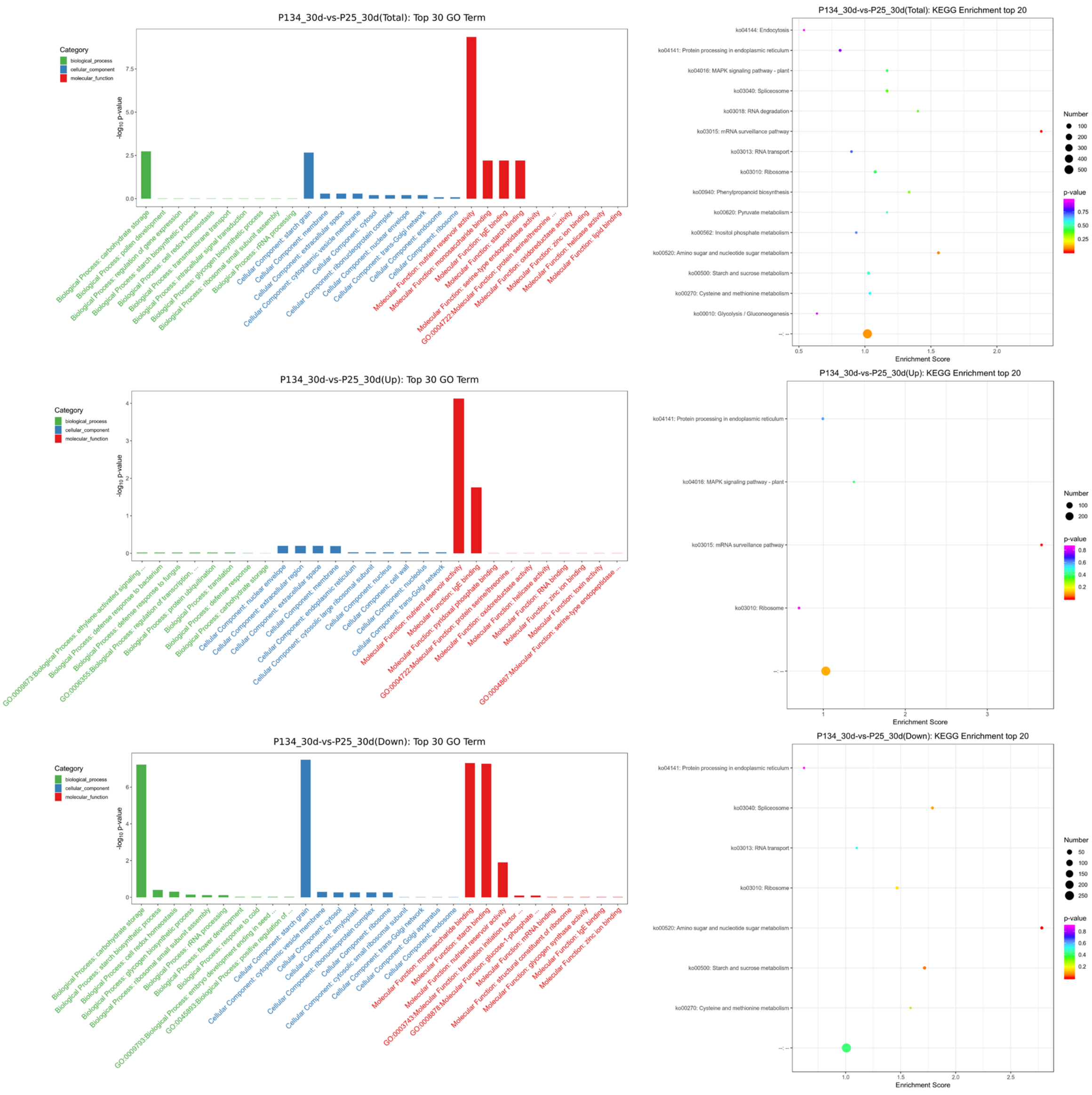

Figure S16. P134_ 30d vs P25_ 30d whole Transcriptome differential circRNA GO and KEGG enrichment analysis (a) P134_ 30d vs P25_ 30d differential expression of circRNA GO enrichment and KEGG enrichment analysis; (b) P134_ 30d vs P25_ 30d differential expression upregulated circRNA GO enrichment and KEGG enrichment analysis; (c) P134_ 30d vs P25_ 30d differential expression downregulated circRNA GO enrichment and KEGG enrichment analysis.

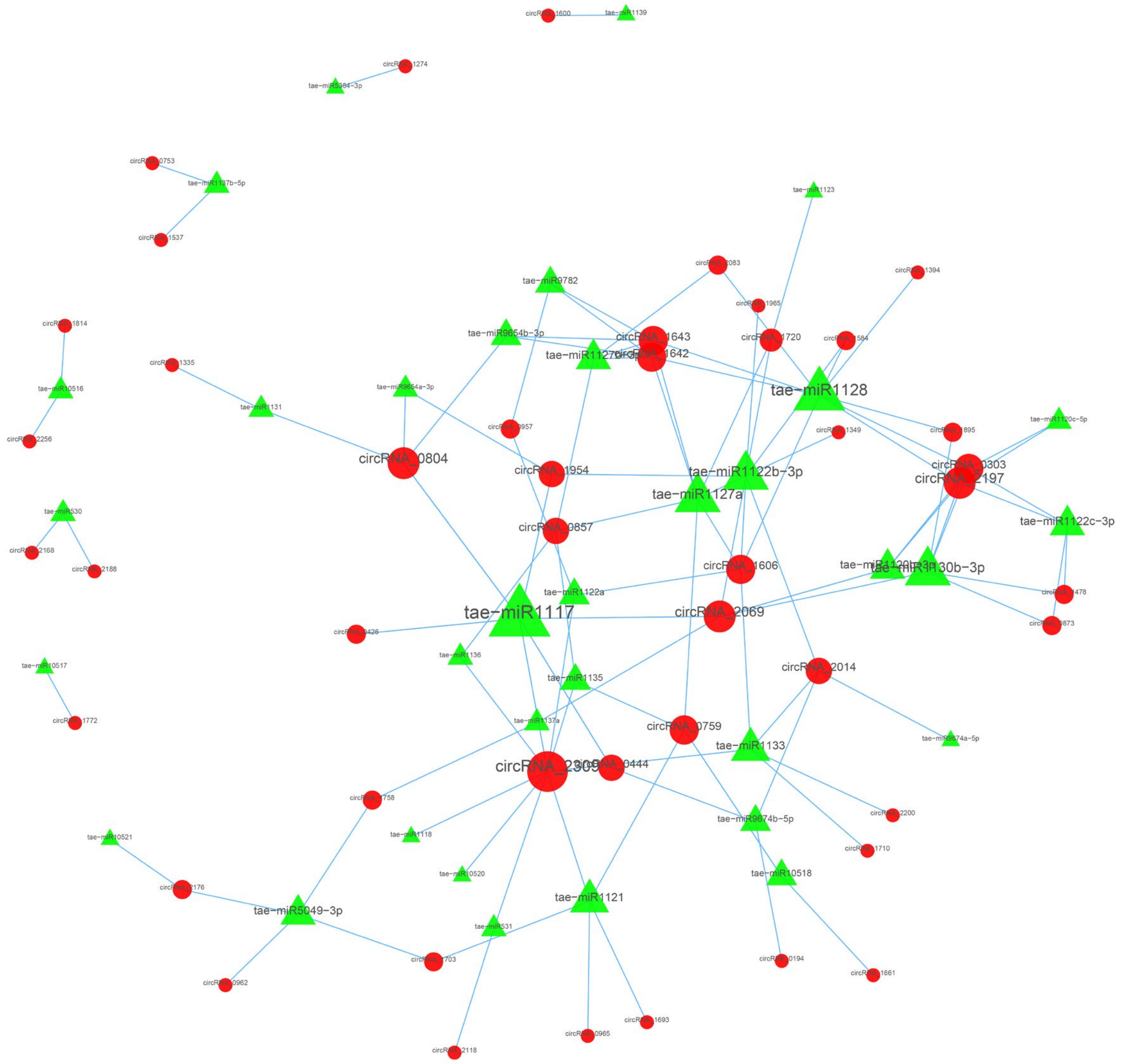

Figure S17. CircRNAs Interacting with miRNAs in Differential Expression of P25_20d vs P25_10d.

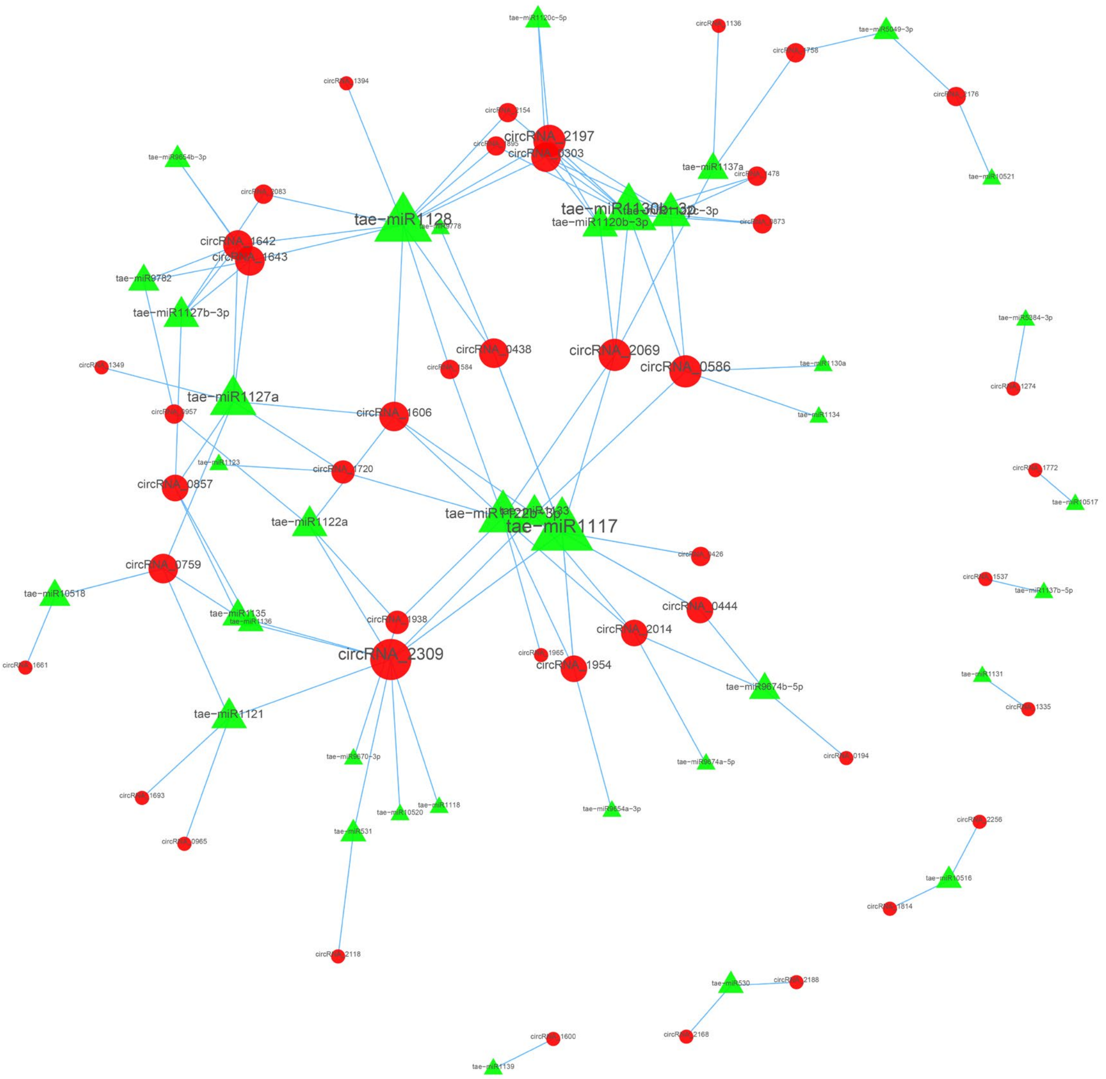

Figure S18. CircRNAs Interacting with miRNAs in Differential Expression of P25_30d vs P25_10d.

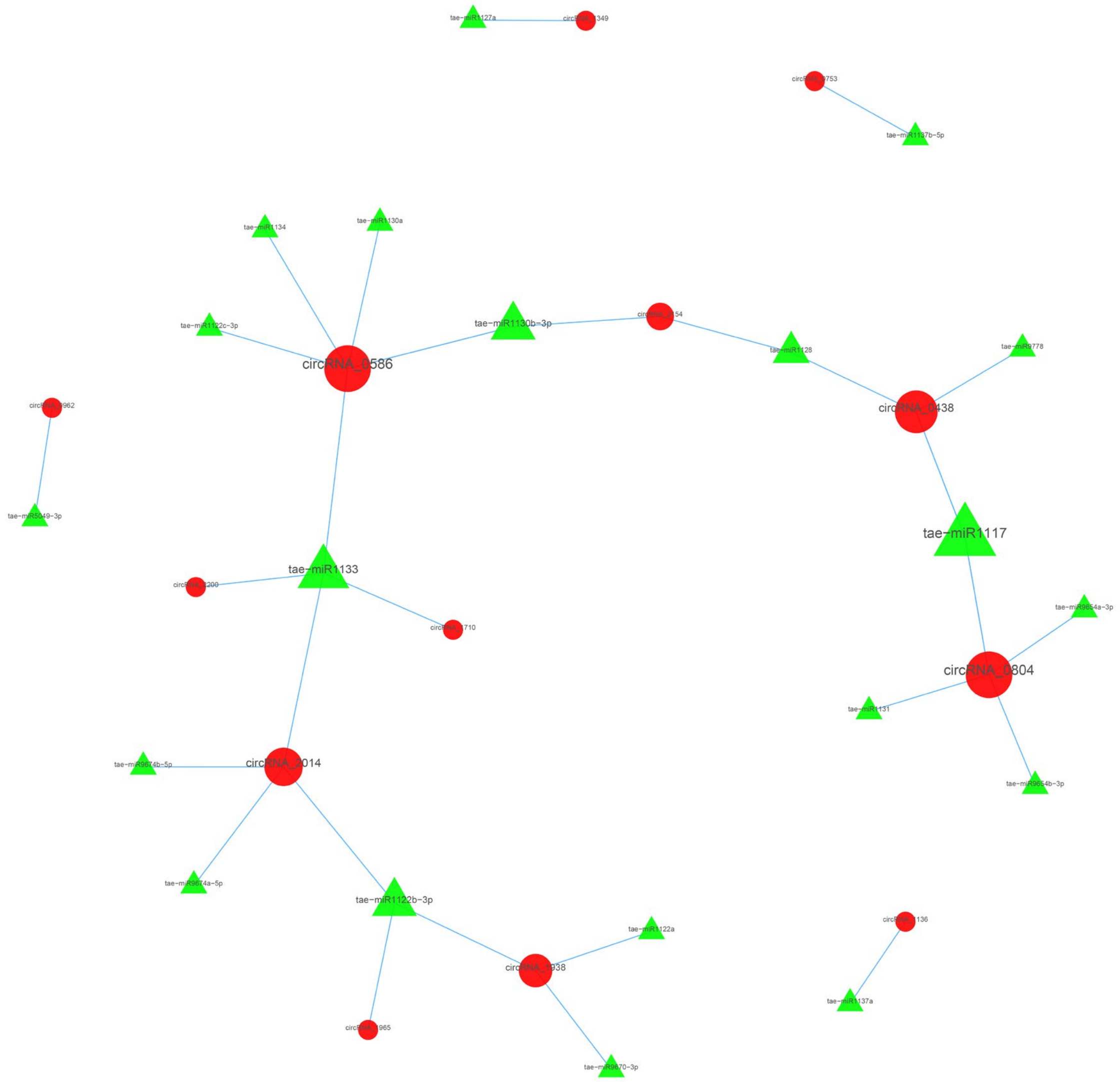

Figure S19. CircRNAs Interacting with miRNAs in Differential Expression of P25_30d vs P25_20d.

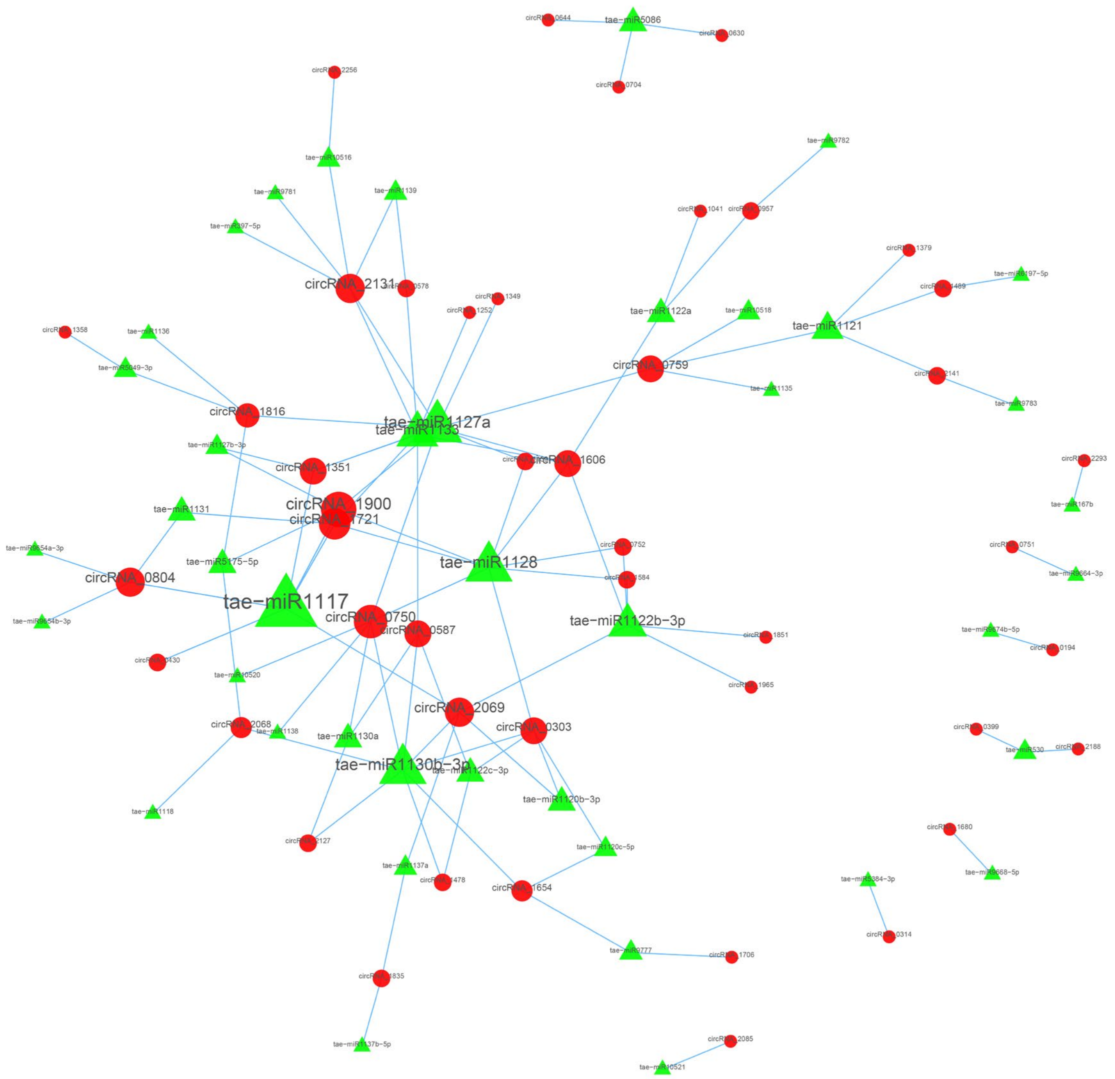

Figure S20. CircRNAs Interacting with miRNAs in Differential Expression of P134_30d vs P134_10d.

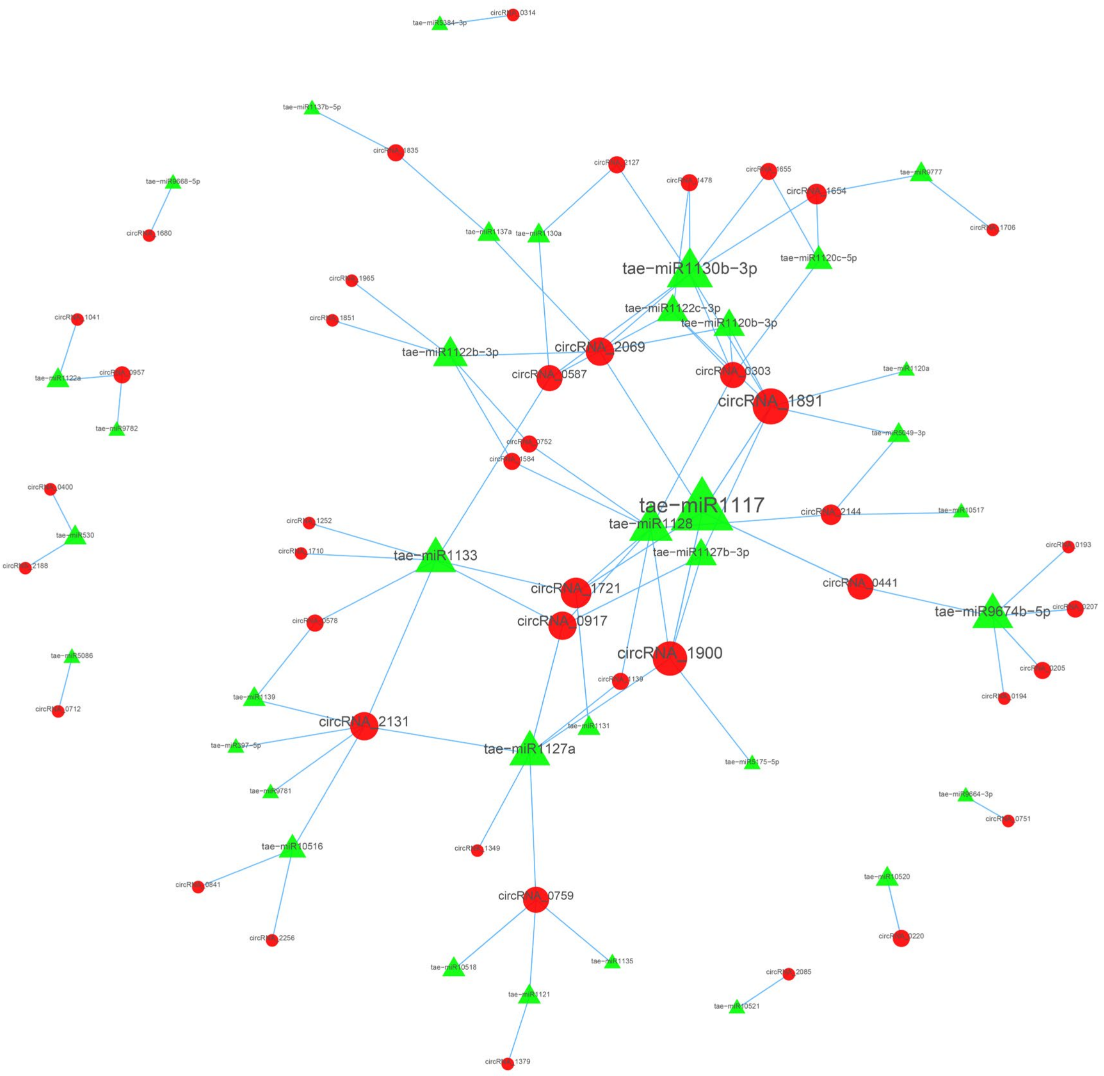

Figure S21. CircRNAs Interacting with miRNAs in Differential Expression of P134_20d vs P134_10d.

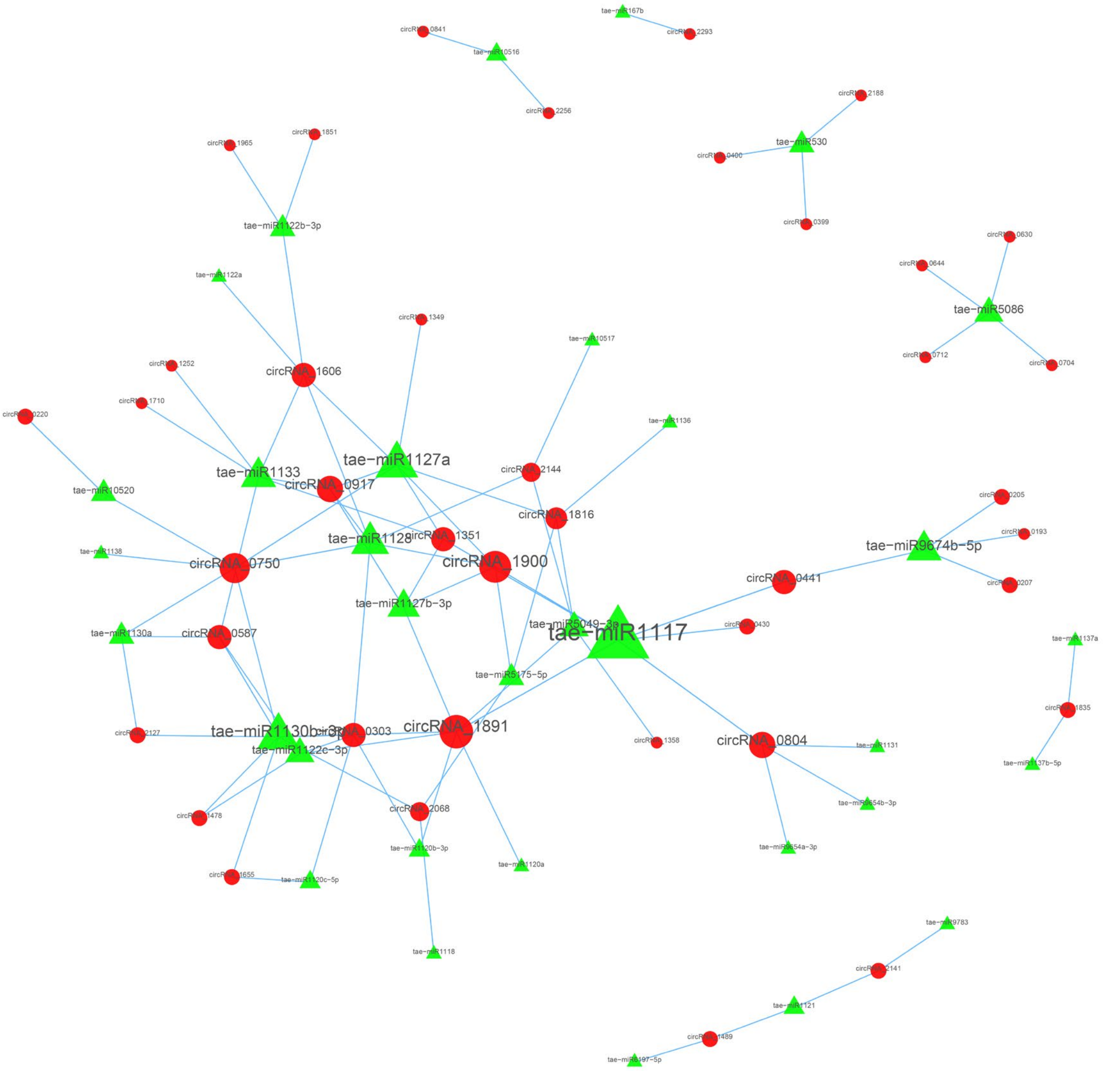

Figure S22. CircRNAs Interacting with miRNAs in Differential Expression of P134_30d vs P134_20d.

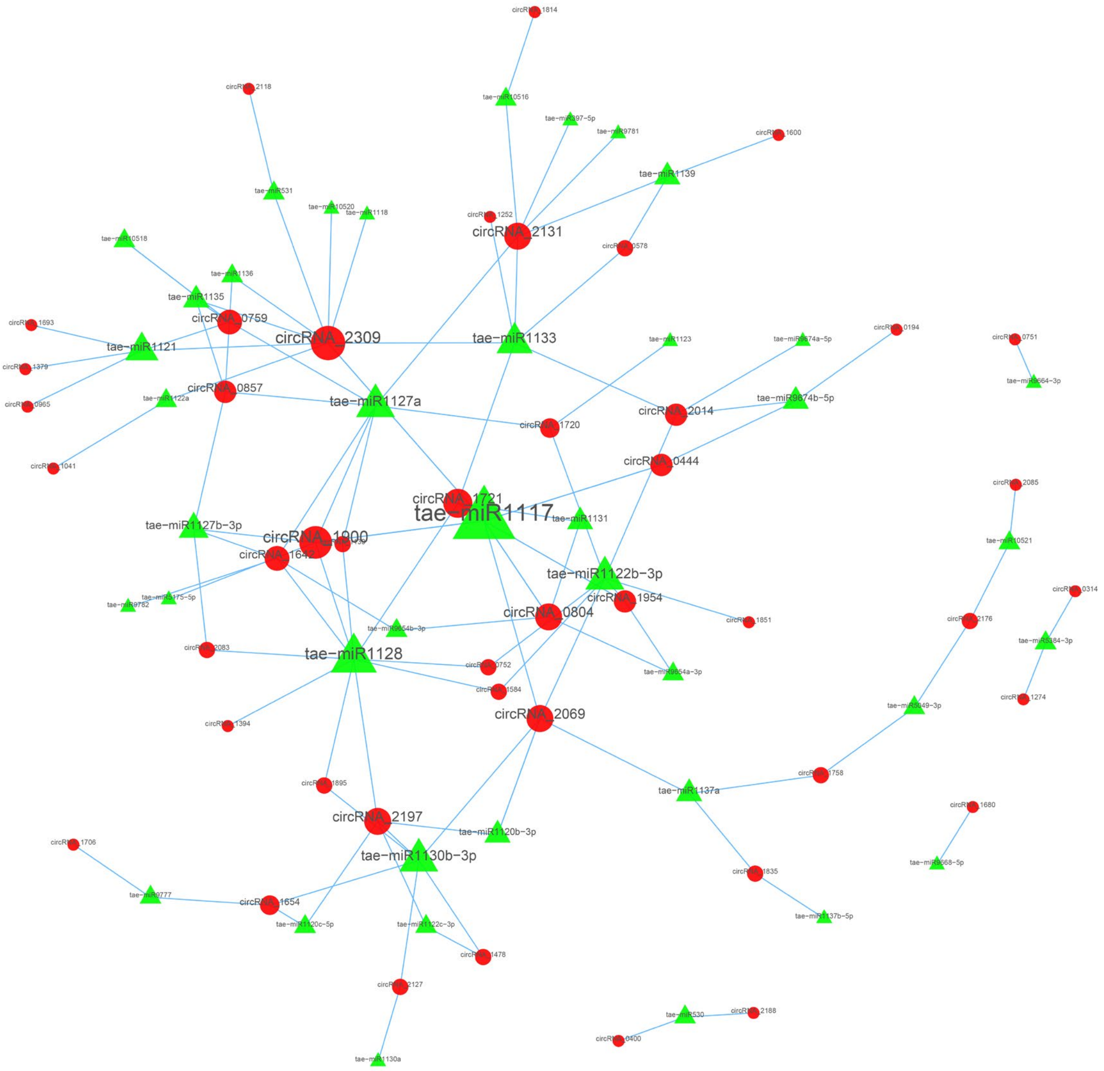

Figure S23. CircRNAs Interacting with miRNAs in Differential Expression of P134_10d vs P25_10d.

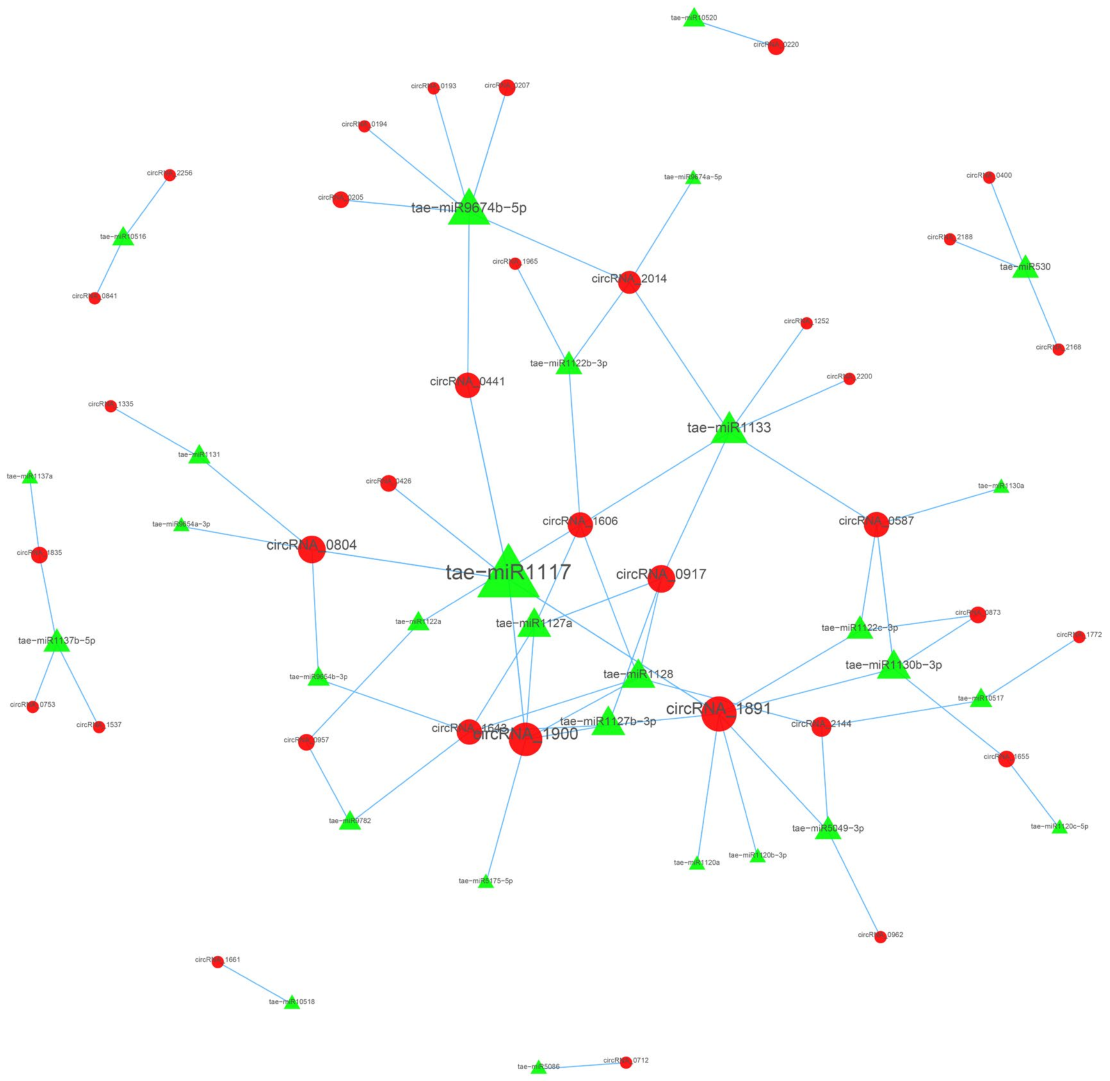

Figure S24. CircRNAs Interacting with miRNAs in Differential Expression of P134_20d vs P25_20d.

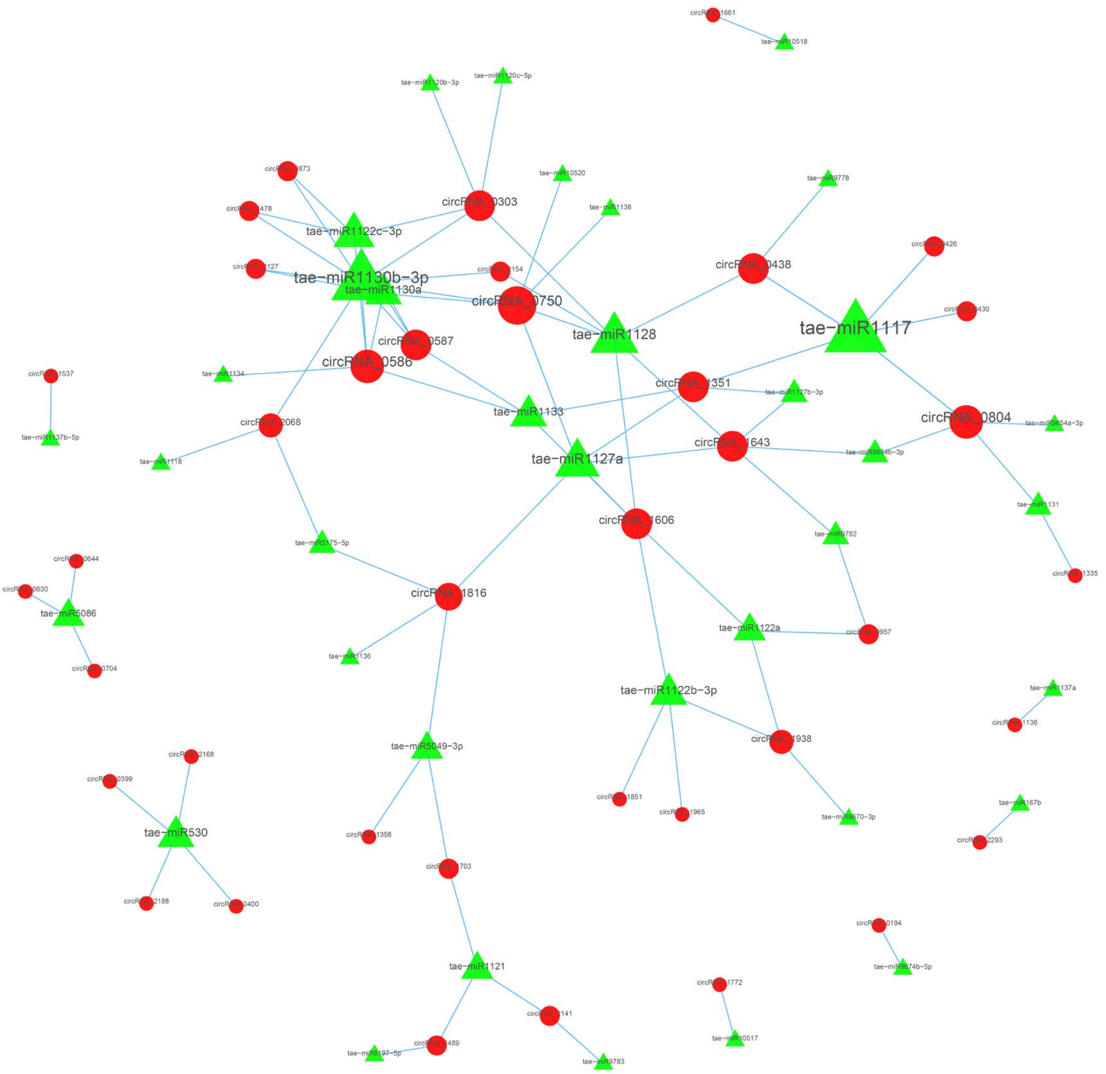

Figure S25. CircRNAs Interacting with miRNAs in Differential Expression of P134_30d vs P25_30d.
